# Malignant Precursor Cells Pre-Exist in Human Breast DCIS and Require Autophagy for Survival

**DOI:** 10.1371/journal.pone.0010240

**Published:** 2010-04-20

**Authors:** Virginia Espina, Brian D. Mariani, Rosa I. Gallagher, Khoa Tran, Stacey Banks, Joy Wiedemann, Heather Huryk, Claudius Mueller, Luana Adamo, Jianghong Deng, Emanuel F. Petricoin, Lucia Pastore, Syed Zaman, Geetha Menezes, James Mize, Jasbir Johal, Kirsten Edmiston, Lance A. Liotta

**Affiliations:** 1 Center for Applied Proteomics and Molecular Medicine, George Mason University, Manassas, Virginia, United States of America; 2 AGTCenter of Genetics & IVF Institute, Fairfax, Virginia, United States of America; 3 Cancer Translational Research Center, Inova Fairfax Hospital, Inova Health System, Falls Church, Virginia, United States of America; 4 Department of Pathology, Istituto Oncologico del Mediterraneo, Viagrande, Catania, Italy; 5 Department of Pathology, Inova Fairfax Hospital, Inova Health System, Falls Church, Virginia, United States of America; Hong Kong University, Hong Kong

## Abstract

**Background:**

While it is accepted that a majority of invasive breast cancer progresses from a ductal carcinoma in situ (DCIS) precursor stage, very little is known about the factors that promote survival of DCIS neoplastic cells within the hypoxic, nutrient deprived intraductal microenvironment.

**Methodology and Principal Findings:**

We examined the hypothesis that fresh human DCIS lesions contain pre-existing carcinoma precursor cells. We characterized these cells by full genome molecular cytogenetics (Illumina HumanCytoSNP profile), and signal pathway profiling (Reverse Phase Protein Microarray, 59 endpoints), and demonstrated that autophagy is required for survival and anchorage independent growth of the cytogenetically abnormal tumorigenic DCIS cells. *Ex vivo* organoid culture of fresh human DCIS lesions, without enzymatic treatment or sorting, induced the emergence of neoplastic epithelial cells exhibiting the following characteristics: a) spontaneous generation of hundreds of spheroids and duct-like 3-D structures in culture within 2–4 weeks; b) tumorigenicity in NOD/SCID mice; c) cytogenetically abnormal (copy number loss or gain in chromosomes including 1, 5, 6, 8, 13, 17) compared to the normal karyotype of the non-neoplastic cells in the source patient's breast tissue; d) *in vitro* migration and invasion of autologous breast stroma; and e) up-regulation of signal pathways linked to, and components of, cellular autophagy. Multiple autophagy markers were present in the patient's original DCIS lesion and the mouse xenograft. We tested whether autophagy was necessary for survival of cytogenetically abnormal DCIS cells. The lysosomotropic inhibitor (chloroquine phosphate) of autophagy completely suppressed the generation of DCIS spheroids/3-D structures, suppressed *ex vivo* invasion of autologous stroma, induced apoptosis, suppressed autophagy associated proteins including Atg5, AKT/PI3 Kinase and mTOR, eliminated cytogenetically abnormal spheroid forming cells from the organ culture, and abrogated xenograft tumor formation.

**Conclusions:**

Cytogenetically abnormal spheroid forming, tumorigenic, and invasive neoplastic epithelial cells pre-exist in human DCIS and require cellular autophagy for survival.

## Introduction

While the transition from in situ to invasive cancer is central to the origin of the malignant phenotype, very little is known about the time of onset, and the triggering mechanism, that switches in situ neoplastic lesions to overt invasive carcinoma in the human breast. Ductal Carcinoma In Situ (DCIS), the most common type of non-invasive breast cancer in women, is defined as a proliferation of neoplastic epithelial cells within the duct that is normally surrounded by myoepithelial cells and an intact basement membrane [1]–[3]. Between 1980 and 2001, the incidence rate of DCIS increased 7.2-fold, presumably due to increasing compliance and improved detection by mammography [1], [3]. DCIS now accounts for an estimated 30% of the 185,000 breast cancers detected by mammography each year [4], [5]. There is both clinical and experimental evidence to suggest that DCIS is a precursor lesion to most, if not all, invasive carcinoma. It is generally accepted that women diagnosed with DCIS remain at high risk for subsequent development of invasive carcinoma, with lesion size, degree of nuclear atypia and the presence of comedo necrosis being histopathological factors of DCIS identified as affecting this risk of recurrence [6], [7]. The critical unanswered biologic questions, addressed in this study, are: Do invasive, cytogenetically abnormal neoplastic cells pre-exist in the pure intraductal DCIS lesion prior to the overt histologic transition to invasive carcinoma? If such precursor carcinoma cells pre-exist in DCIS, does autophagy support their survival in the face of nutrient deprivation and hypoxia?

It has been previously hypothesized that breast cancer progression is a multi-step process involving a continuum of changes from the normal phenotype to hyperplastic lesions, carcinomas in situ, invasive carcinoma, and finally to metastatic disease [8]. Under this model additional genetic alterations are required before neoplastic cells in a DCIS lesion can progress to an invasive and metastatic carcinoma. However, more recent refinements of this model indicate that the aggressive phenotype of breast cancer is determined at the premalignant stage, much earlier than previously thought. Experimental approaches employing loss-of-heterozygosity (LOH), and comparative genomic hybridization (CGH) provide strong evidence that DCIS and invasive carcinomas in the same patient share similar genetic alterations [6], [7], [9], [10]. Gene expression studies of patient-matched tissues including atypical ductal hyperplasia (ADH), DCIS, and invasive carcinoma revealed that the various stages of disease progression are very similar to each other at the level of the transcriptome [7], [9], [10]. These studies also show that the DCIS lesions at the level of gene expression are more similar to the invasive cancers in the same patient compared to DCIS lesions in other patients [7], [10]. Damonte *et al* employing the ‘MINO’ (mammary intraepithelial neoplasia outgrowth) mouse model of DCIS concluded that malignant aggressiveness is pre-programmed in the pre-cancer stem cell [6]. Taken together, these data support the hypothesis that the invasive phenotype of breast cancer is already programmed at the pre-invasive stages of disease progression.

In the present study we studied the cellular processes that promote the survival of the invasive precursor cells that exist within the human breast intraductal niche. Living human DCIS ducts were explanted into organoid culture, in serum free conditions, to expose the putative DCIS neoplastic cells within the duct. We characterized the biologic phenotype of these emerging cells using xenograft transplantation, and propagation in organ culture and evaluated the survival mechanisms employed by these cells. Cytogenetically abnormal DCIS lesion derived epithelial cells were identified by their phenotype of anchorage independent growth as 3-D spheroids and duct like structures, invasion of autologous stroma, and tumorigenicity in SCID NOD mice. We find that autophagy is a principal, and perhaps necessary, survival pathway in DCIS.

To date, the macroautophagy survival pathway (autophagy) has not been implicated in the survival of malignant precursor cells within human DCIS lesions. Autophagy (self-eating) is a dynamic, catabolic process of organelle digestion that generates ATP during periods of nutrient limitation [11]–[13]. Autophagy optimizes nutrient utilization in growing cells faced with hypoxic or metabolic stresses (Figure S1). During autophagy, macroautophagosomes (also referred as autophagosomes) are formed as double membrane-bound vesicles that engulf cytoplasmic constituents and/or cytoplasmic organelles. Autophagosomes fuse with lysosomes to degrade the contents of the autophagic vesicle and provide molecular break down products to either feed the cell or enable cell death. Accumulation of autophagosomes can be due to reduced turnover of autophagosomes or increased autophagic activity [14]. Cancer cells may undergo autophagic cell death associated with extreme autophagic degradation after exposure to several cancer therapies [15]. While some initial studies described autophagy as a tumor suppressor mechanism [16], the autophagic response can also function as a protective mechanism allowing the recycling of proteins and cellular components to facilitate cell survival during the severe cellular stress of cytotoxic therapy [17]. It remains largely unknown whether “protective autophagy” might also sustain the survival of premalignant or malignant progenitor cells with the intraductal environment of the DCIS lesion. Proliferating DCIS epithelial cells accumulating within the breast DCIS lesion are separated from the surrounding lymphatic and vascular supply by the ductal epithelial basement membrane. High grade DCIS is associated with central necrosis in the duct, and the accumulation of cellular degradation products such as lipofuschin. Autophagy is a plausible means for DCIS neoplastic cells, accumulating in the duct, to survive in the face of severe metabolic, oxidative, and hypoxic stress. We directly tested whether autophagy was necessary for the survival of cytogenetically abnormal, tumorigenic DCIS cells by treating the DCIS cells with the lysosomotropic inhibitor (chloroquine phosphate).

## Results

### Human DCIS cells are tumorigenic in NOD/SCID mouse xenografts

**Figure 1:**
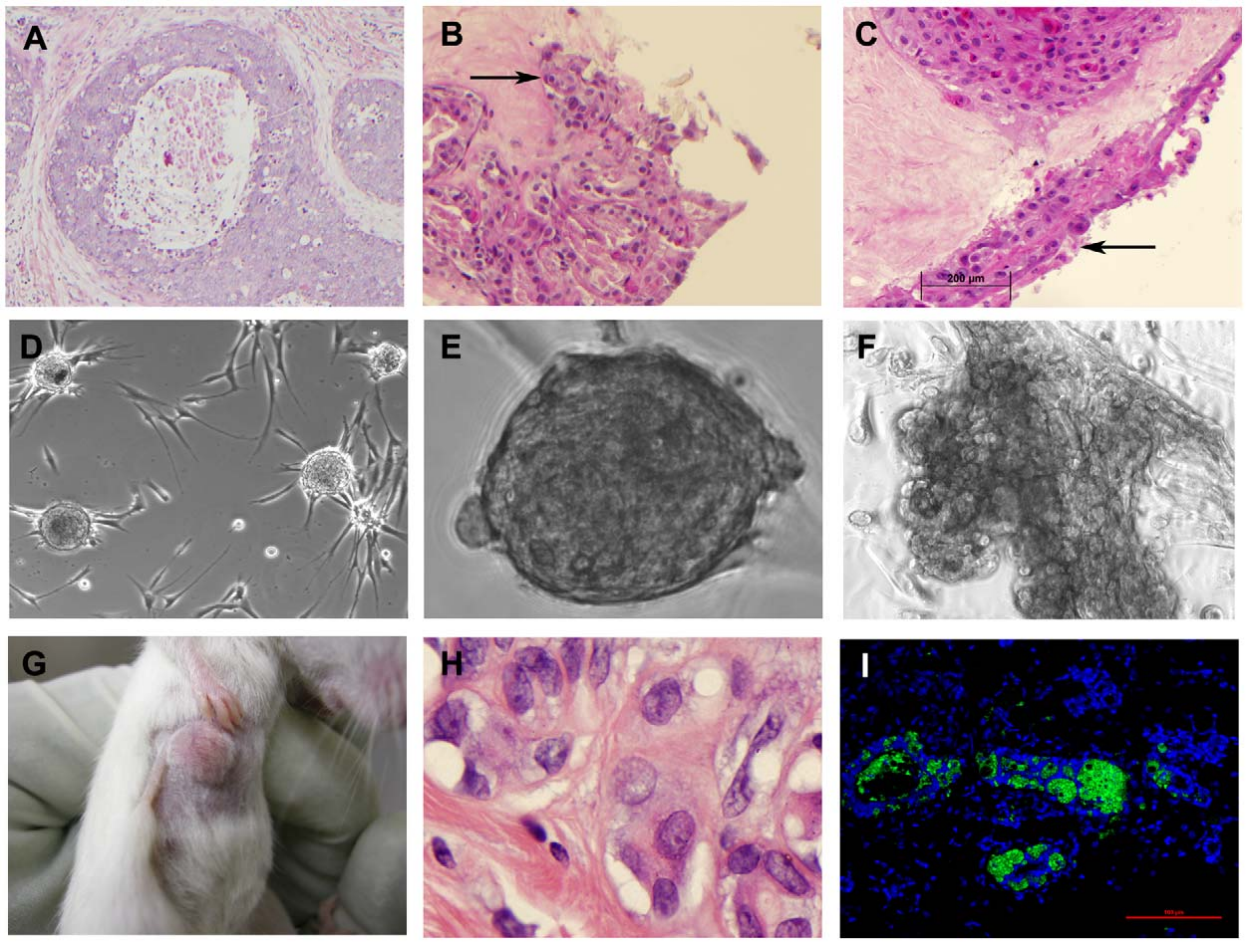
Human DCIS tissue generates spheroids and pseudoductal structures in *ex vivo* culture and xenograft neoplasms. (A) H&E stain of human breast DCIS, grade III with comedo necrosis (case 08-352), that represents the primary surgical source material for our organoid culture model system (10×). (B) DCIS cultured neoplastic cell exhibited autologous stromal invasion (20×). (C) H&E stain of multi-layered pleomorphic epithelial cells growing on the surface of autologous breast stroma after 12 weeks in culture (20×). Organoid culture of human DCIS lesions in serum free conditions spontaneously yielded (D) epithelial spheroids (10×) with a single spheroid shown in (E) (40×) and (F) pseudoductal structures with lumen formation (20×). (G) Human DCIS organoids or spheroids transplanted in NOD SCID mice induced tumor formation at the mammary fat pad transplantation site within 2 months. Xenograft tumors were greater than 16 mm^2^ (length x width) as measured with calipers. (H) H&E stain of murine xenograft tumor (100×). Note pleomorphic epithelial cells with prominent nucleoli, stromal invasion and partial glandular differentiation. (I) Murine xenograft tumors derived from human DCIS cells were shown to be of human origin by the presence of human-specific epithelial antigen in formalin fixed paraffin embedded tissue sections. EpCAM-FITC (pseudo-colored green, 488 nm) and DAPI (psuedo-colored blue, 408 nm) (20× objective).

The existence of tumorigenic cells within fresh human DCIS duct segments was first tested using xenotransplantation [18]. Multiple independent xenograft transplants of human DCIS organoids, with no histological evidence of invasive carcinoma, generated tumors within 1 to 2 months in NOD SCID mice. Xenograft transplants generated from invasive breast carcinoma or DCIS with invasive components were used as positive controls. The number of positive control tumors observed for mixed DCIS or invasive breast carcinoma tissue was 9 of 20 transplanted (45%) (data not shown). 56 mice were transplanted with either freshly procured DCIS duct segments, propagated xenograft tumors, or organoids and/or human primary cultured cells generated from the DCIS tissue (Figure 1). The number of tumors generated within 3 months of injection was 43/56. The number of tumors observed for pure DCIS tissue, which included cribriform DCIS, as well as DCIS Grades II and III, was 18 of 22 transplanted (81%). Xenograft propagated tumors, which were derived initially from pure DCIS tissue, produced tumors in 4 out of 5 mice for 2 generations (Figure 1 and Figure S2).

In order to identify and characterize the tumorigenic neoplastic cells within the DCIS tissue that were responsible for the tumorigenic phenotype observed in the mouse xenografts, we cultured fresh living human DCIS ductal fragments to generate DCIS neoplastic cell outgrowths. The outgrowths spontaneously generated 3-D structures including spheroids and duct-like structures (Figure 1D–F). The number of tumors observed from cultured primary cells and/or spheroids was 21 of 27 transplanted (77.7%). Xenograft tumors arising from pure DCIS tissue contained partially formed ductal structures with stromal infiltration and were positive by immunofluorescence (IF) for human specific epithelial cell adhesion molecule (EpCAM) (Figure 1G–I). No evidence of organ metastasis was noted at necropsy. These data clearly demonstrate that both DCIS tissue and cultured DCIS spheroid structures were capable of inducing tumors with comparable tumorigenic potential.

### Anchorage independent neoplastic epithelial cells spontaneously emerge in organ culture of human DCIS

Organoid culture was used to characterize the DCIS neoplastic cells that were implicated in tumorigenesis by the xenograft experiments. Migratory proliferative cells that were positive for human specific EpCAM were observed to migrate out of the DCIS duct organoids grown in culture for as little as 2 weeks (within 2–4 weeks). Continued *in vitro* organoid cultivation successfully propagated DCIS derived epithelial cells with anchorage independent growth, defined as upward growing and expanding spheroids, and lobulated, duct-like 3-D formations with pseudo lumens, in serum free medium supplemented with EGF and insulin (Figure 1).

DCIS cultured neoplastic epithelial cells migrated over the surface of autologous stroma and formed multilayered colonies with clear epithelial morphology (Figure 1C). Invasive foci beneath these outgrowths within autologous stroma were verified by absence of type IV collagen basement membrane. Seven human DCIS derived epithelial strains, capable of generating spheroids, have been propagated and characterized to date, some for as long as one year (Table 1). Sub-passage of DCIS organoids reconstituted the 3-D ductal and spheroid phenotypes, which reproducibly invaded inward from the surface of autologous stroma in organoid culture. The culture conditions consistently generated a high yield of DCIS epithelial cell outgrowths for each patient, even with small volumes of starting tissue.

**Table 1:** Patient characteristics for generation of *ex vivo* DCIS cultures.

Sample	Age	Pathologic Diagnosis	Morphologic subtype	Nuclear Grade	ER (% positive)	PR (% positive)	Time in ex vivo culture (months)
08-183	47	DCIS	CN/CR	3	30%	Neg	6
08-352	42	DCIS	CR-extended	3	50%	50%	12
09-091	68	DCIS/ADH	CR	2/3	+	+	8
09-118	49	ADH*	Stromal fibrosis-AH	2	+	N/A	8
09-148	45	DCIS	Solid and CR	3	90%	90%	7
09-301	34	DCIS	Solid and CR	2	90%	90%	4
09-327	57	DCIS	Cribriform with IP	2	+	+	2

DCIS = ductal carcinoma in situ; ADH = Atypical ductal hyperplasia; ER = Estrogen Receptor; PR = Progesterone Receptor; + indicates positive result, no cell percentage specified;

* Previous history of DCIS, patient treated with Tamoxifen citrate; CN = Comedo necrosis; CR = Cribriform; CR-extended  =  extension into lobules with no evidence of invasion; AH = Pseudoangiomatoid hyperplasia; IP = Intraductal papilloma.

### DCIS derived tumorigenic spheroid forming cells are cytogenetically abnormal

The genotype of the isolated DCIS spheroid forming cells was evaluated and compared to the non-neoplastic tissue from the same patient. The spheroid forming DCIS epithelial cells were found to be cytogenetically abnormal compared to the normal karyotype of the anchorage dependent monolayer cells cultured from the same patient's breast tissue lesion. Full genotypic data output included allele calls from “tagged” single nucleotide polymorphism (SNP) sites and signal intensity values from non-polymorphic sites to determine DNA copy number values (CytoSNP-12 beadchips (Illumina, Inc.). Molecular cytogenetic profiles demonstrated cytogenetic alterations in the isolated DCIS spheroids (3–5 spheroids per prep) and isolated pseudoductular structures. The spheroid abnormal karyotype signature included loss of copy number on chromosome 5, 6, 8, and 13, and gain of copy number on chromosomes 1, 5, and 17. Abnormalities were present in all DCIS cell spheroids and pseudoductular isolates (Table 2) and arose in a background of cells with a normal karyotype from the same patient.

**Table 2:** Molecular karyotype of DCIS spheroid cultures showing cytological location of chromosome aberrations.

Sample ID	Gain* of DNA	Loss* of DNA	Copy-neutral LOH
08-352	1p36.31p13.2	2q31.1q32.1	6p21.33p21.32
	1q21.1q41	4pterp15.2	12pterp12.1
	2(triploid)	8pterp12	12q11.22
	3q11.2	11q14.1q22.1	13q11q33.1
	6q22.31q25.1	14^b^	17pterp13.1
	8q22.3q24.3	17^b^	Xq13.1q23
	9p22.3p22.1	19^b^	
	11q22.1q23.1	Xq25	
	12p11.21q12		
	13q33.2qter		
	14^a^		
	15q25.2qter		
	17^a^		
	18(triploid)		
	19^a^		
	20q13.13q13.2		
	Xq23q26.2		
09-091	8(triploid)	6q21, 6q16.1	22q11.21q11.22
	10q22.3	13q21.31q21.33	
	22q11.23q12.1	21q21.1q21.2	
09-118		5p15.2, 5p15.1p13.3	3q28q29
		10q21.3	5p15.32, 5p15.2p15.1, 5p13.3
		11p11.2p11.12	5q14.1
		12p13.1p12.3	6q22.31q23.3, 6q25.3q26
		12p12.1p11.22	10q21.1q21.2, 10q22.1q22.3, 10q24.1q26.3
		12q12q13.11	12^c^
		15q21.1q21.3	14q31.1-q32.11, 14q32.13q32.2
		18p11.32	15q14q21.1, 15q22.1q22.2
			16p13.3p13.2
			17q25.3
			22q11.23q13.31
			Xp22.33p22.11
			Xq22.1q28
09-148	1q21.1, 1q21.3	5p14.3, 5p14.1	6q22.31
	1q32.1q32.3	6q14.1, 6q24.1	22q13.31qter
	5p15.2, 5p13.3p12	8q2.3, 8q22.3	Xq22.1q22.3, Xq22.3, Xq23
	17q12, 17q21.32q21.33	8p23.2p23.3	
	17q22q23.3	13q21.1	
	20q13.31qter		
09-301			2q21.3q22.1
			4q31.21q31.22
			5q21.3q22.1, 5q23.331.1
			8q23.2
			14q23.2q23.3

Cytological locations are listed as chromosome:arm:band(s);

* Gain (3 or more copies) or Loss (0 or 1 copy) of DNA >3 million base pairs; LOH = loss of heterozygosity.

a multiple regions of DNA gain;

b multiple regions of DNA loss;

c multiple regions of copy neutral (diploid) loss of heterozygosity.

Table 2 summarizes gain (3 or more copies) or loss (0 or 1 copy) of DNA >3 million base pairs. If gains or losses greater than 1 million base pairs were used as the cutoff, then, at this higher resolution, anchorage independent spheroid cells from 3 different patient DCIS lesions all showed narrow copy number loss of chromosome 6 (p21.1/p12.3). This region includes the transcription factor SUPT3H (protein coding GIFtS:59, GC06M044904, UniProtKB/Swiss-Prot: SUPT3_HUMAN, O75486) (Figure S3). A second region of aberration was observed in a single patient on the p-arm of chromosome 5 entailing extended regions of gain and loss of chromosomal content. Chromosomal bands from 5p12 to 5p13.3 were present in 3 copies and a distal segment of 5p13.3 included 4 copies. Bands 5p14.1 and 5p14.3 on the same chromosome however showed loss of DNA content as represented by homozygous and hemizygous deletions, respectively (Figure S4). This same patient's cultured DCIS cells showed a 14 Megabase (Mb) region of trisomy on chromosome 17, extending from 17q22 to 17q25.1 (Figure S5). The SNP data indicated, for all matched samples, that the DCIS cultured cells were derived from the donor patient tissue and were not a contaminating cell line. The normal epithelial and stromal cells of the donor tissue grown for the same length of time in culture possessed a fully normal karyotype, while in all cases the propagated DCIS cells forming 3-D structures and exhibiting invasion exhibited genetic copy number gain or loss derangements in one or more genetic loci indicative of a neoplastic mutational event (Table 2, Figures S3-S6). Multiple isolates from different regions of the DCIS lesion, for the same patient, yielded spheroids with the same abnormal molecular karyotype. Thus, cytogenetically abnormal, DCIS derived, spheroid forming epithelial cells, emerged spontaneously from organoids in culture and were responsible for the tumorigenic and invasive phenotype observed (Figure 1).

### Signal pathway proteomic analysis reveals augmentation of survival related pathways

Within the organoid cultures we sought to characterize the functional signal pathways that may be involved in the phenotype of these cells. It would not be possible to measure a large number of protein signal pathway endpoints and post-translational modifications by conventional flow cytometry following enzymatic dissociation, even within a hundred spheroids. Consequently we used Reverse Phase Protein Microarray (RPMA) analysis of 59 cell signaling kinase endpoints, representing stem cell markers, autophagy, adhesion, invasion, and pro-survival pathways (Table S1). RPMA technology has the required sensitivity and precision for small numbers of cells and provides a means of quantifying phosphoproteins indicative of activated signal pathways [19], [20].

**Figure 2:**
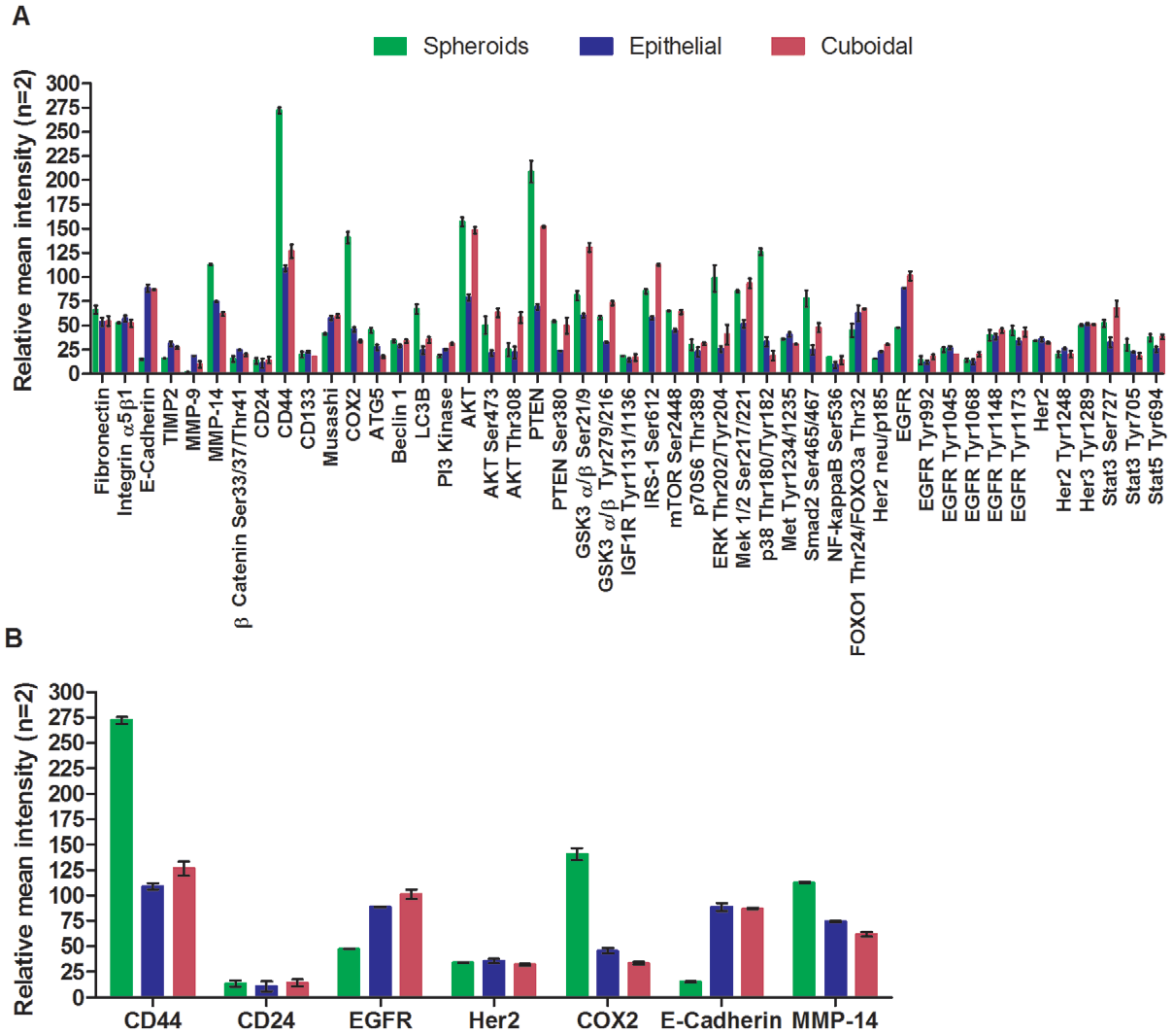
Signal pathway mapping of DCIS organoid outgrowths by reverse phase protein microarray. The activation state of signaling pathways in the DCIS spheroids was compared to the anchorage dependent cells in organoid culture to characterize the cell populations. 48 endpoints were analyzed representing total or post-translationally modified proteins. Under direct microscopic visualization we procured spheroids, the epithelial monolayer, and separate cuboidal monolayer cells from the same DCIS organoid culture. Approximately 25 spheroids were analyzed. Data was normalized to β-Actin per microarray spot as described in VanMeter et al [19]. (A) Comprehensive depiction of cell signaling kinase activity for spheroids (green), epithelial (blue) and cuboidal (red) cell populations (relative mean intensity, n = 2). Autophagy markers (Atg5 and LC3B) were elevated in the spheroids in comparison to the epithelial and cuboidal monolayer cells. p38 MAPK Thr180/Tyr182 and SMAD2 Ser465/467, cell signaling proteins associated with survival and stress were elevated in the spheroids in comparison to the epithelial and cuboidal monolayers. (B) The spheroids exhibited progenitor cell characteristics as evidenced by augmentation of CD44, COX-2, and matrix metalloproteinase (MMP-14), with associated reduction of E-Cadherin. The spheroids were found to be cytogenetically abnormal (Figures S4–S6) compared to the normal karyotype of the epithelial monolayer.

Comparison of the spheroids to the flat, single layer epithelial cells in the same culture revealed a set of activated signaling pathways consistent with a progenitor-type classification. Autophagy markers (Atg5 and LC3B) were elevated in the spheroids in comparison to the epithelial and cuboidal monolayer cells. p38 MAPK Thr180/Tyr182 and SMAD2 Ser465/467, cell signaling proteins associated with survival and stress, were elevated in the spheroids in comparison to the epithelial and cuboidal monolayers. The spheroids exhibited progenitor cell characteristics as evidenced by up-regulation of stem cell markers (CD44), down-regulation of cell adhesion markers (E-Cadherin), up-regulation of invasion related metalloproteinases (MMP14), and up-regulation of COX-2 (Figure 2).

To test whether these differences in cell signaling proteins were a stable characteristic of the observed phenotype, we conducted an independent verification analysis. RPMA was performed on a different set of harvested cultured spheroids, epithelial cells, and stromal fibroblasts from the same patient derived cultures that were propagated over several months (Table 1). Autophagy, adhesion, and pro-survival signaling proteins remained elevated in the spheroids compared to the epithelial and fibroblast cells (Table S2).

### Autophagy is elevated in DCIS cells

**Figure 3:**
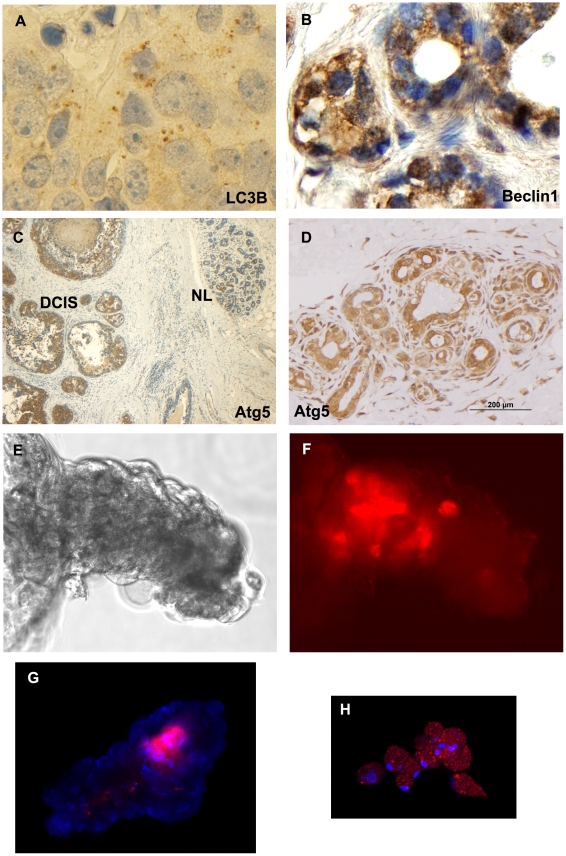
Autophagy is activated in human DCIS lesions and xenograft tumors and is inhibited with chloroquine. Immunohistochemistry markers of autophagy were examined in primary DCIS lesions, mouse xenograft tumors, and DCIS *ex vivo* generated spheroids/pseudoductal structures. Autophagy markers (Atg5, LC3B, Beclin1) exhibited prominent positive staining in primary DCIS lesions (Table 3). (A) IHC of a primary DCIS lesion showing punctate staining within the cytoplasm for LC3B a protein associated with autophagosome formation (anti-LC3B, 100×). (B) Beclin1 positive human DCIS derived mouse xenograft tissue (100×). (C) Case 08-352 surgical specimen is positive for Atg5 staining in comedo DCIS human breast glands (DCIS) compared to adjacent non-neoplastic ductal elements (NL) (10×). (D) Positive Atg5 staining of a DCIS organoid after 12 weeks in culture (20×). (E–H) Autophagy is also activated in cultured DCIS pseudoductal structures and spheroids. (E) A bright field image of a multi-cellular pseudoductal structure (20×). (F) Fluorescence microscopy shows accumulation of LysoTracker Red dye within the organelles of the inner cell mass of the structure shown in panel E (20×). (G) LysoTracker Red dye accumulation within the central cell mass of a spheroid (red = LysoTracker Red; blue = DAPI nuclear counterstain, 20×) demonstrates enhanced phagosome and lysosomal activity in the region of the aggregate expected to be most hypoxic. (H) Chloroquine inhibits autophagy by preventing the fusion of autophagosomes and lysosomes in the dynamic, multi-step autophagy cascade. An organoid culture was maintained in culture medium supplemented with chloroquine phosphate (50 µM) for 2 weeks. Dissociation of the spheroid, and diffuse accumulation of LysoTracker Red in autophagic compartments and lysosomes were observed (red = LysoTracker Red; blue = DAPI nuclear counterstain, 20×, Nikon Eclipse TE200 microscope).

Based on the RPMA phenotypic characterization, we noted that cell signaling pathways intersecting with the autophagy pathway were up-regulated in the cultured DCIS spheroids and 3-D structures. Autophagy markers were found to be activated in DCIS lesions *in vivo*, DCIS cultured organoids, and murine human DCIS xenografts. Intermediate and high-grade DCIS lesions were positive by immunohistochemistry for autophagy pathway proteins Atg5, Beclin-1 and LC3B, which are involved in the nucleation of autophagosomes (Table 3). Autophagosome accumulation, as demonstrated by fluorescence microscopy and immunohistochemistry of endogenous LC3B, showed an increase in punctate LC3B, a hallmark of autophagy because it is the first protein to associate with the autophagosomal membrane (Figure 3) [14], [21]. The acidotropic dye, LysoTracker Red (Invitrogen), which accumulates in intracellular organelle components associated with autophagy (autophagosomoses/lysosomes), was used to image live DCIS organoids culture cell outgrowths, including spheroids and 3-D structures (Figure S1). In DCIS progenitor cells forming spheroids or invading autologous stroma, lysosomal or autophagosome activity was up regulated in the central region of the spheroid as shown by strong fluorescence with LysoTracker Red (Figure 3F&G) and distinct staining of LC3B and Atg5 by IHC in FFPE tissue sections (Figure 3A & C). This is consistent with the up-regulation of autophagy to promote survival in the hypoxic and nutrient deprived center of the spheroid in culture or the intraductal DCIS microenvironment.

**Table 3:** Immunohistochemical characterization of primary patient breast tissue.

Sample ID	Diagnosis	LC3B	Beclin1	Atg5	CD44
08-183	DCIS	1+	3+	3+	Positive
08-352	DCIS	1+	3+	3+	Positive
09-091	DCIS/ADH	0	1+	1+	Negative
09-118	ADH*	N/A	1+	1+	Positive
09-148	DCIS	0	2+	1+	Positive
09-301	DCIS	0	2+	1+	Positive
09-327	DCIS	0	2+	1+	Negative

DCIS = ductal carcinoma in situ; ADH = Atypical ductal hyperplasia.

* Previous history of DCIS, patient treated with Tamoxifen citrate.

### Autophagy is required for emergence and survival of DCIS tumorigenic spheroid cells

**Figure 4:**
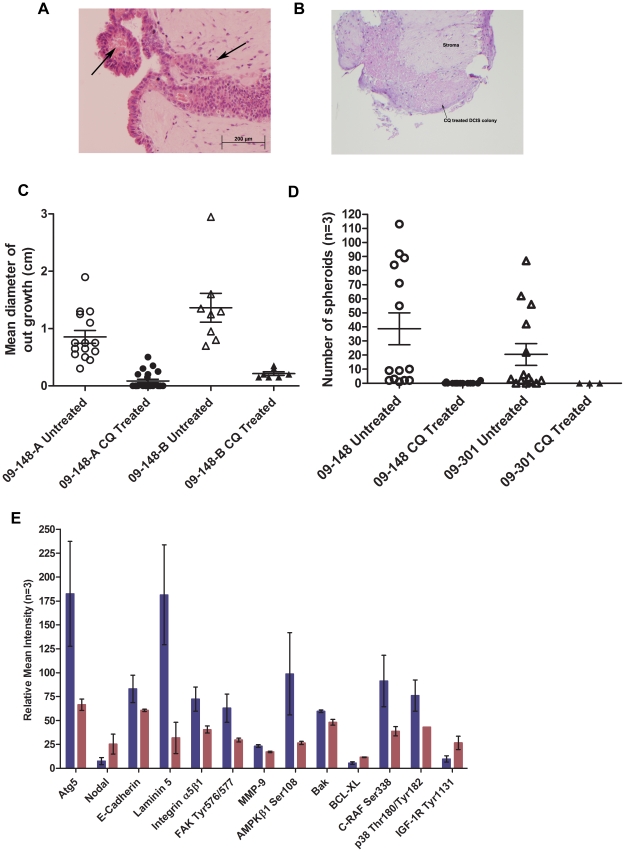
Chloroquine suppresses DCIS neoplastic cell outgrowth and spheroid formation and alters cellular signaling. (A) Untreated DCIS cells grow on the surface of autologous stroma, spontaneously form pedunculated spheroids with an area of central necrosis (arrow), and invade the autologous stroma (arrow). (B) DCIS organoid cultured in the presence of chloroquine for 2 days showed complete absence of cellular outgrowths and degenerated cells within the duct (arrow) (10×). (C) Chloroquine suppressed outgrowth of DCIS epithelial cells in culture as measured by outgrowth diameter. Two axis diameters were measured for multiple organoids for two cases. Case 09-148-A mean diameter outgrowth prior to treatment (open circle) was 0.85 cm±0.11 (n = 15, mean ±SEM) and after chloroquine treatment (black circle), the mean diameter was 0.084 cm±0.03 (n = 23, mean ±SEM) (p<0.0001). Case 09-148-B mean diameter outgrowth prior to treatment (open triangle) was 1.36 cm±0.25 (n = 8, mean ±SEM) while the chloroquine treated outgrowth (black triangle) mean diameter was 0.21 cm±0.03 (n = 7, mean ±SEM) (p = 0.0026). (D) The number of spheroids generated in the untreated cultures (open circle, case 09-148) ranged from 1 to more than 100 for individual duct fragments (mean of 38.7±11; n = 14, mean ±SEM). Following chloroquine treatment, 12 out of 14 explants did not have any spheroids (mean number of spheroids post treatment 0.21±0.15; n = 14; p = 0.0049, black circle, mean ±SEM). For case 09-301, the mean number of spheroids prior to treatment was 20.5±7.8, n = 14 (open triangle, mean ±SEM) and there were no spheroids observed after treatment (n = 3; black triangle, mean ±SEM). (E) Epithelial cells and spheroids were maintained in culture, followed by treatment with chloroquine phosphate (50 µM) for 4 days. Spheroids were harvested pre and post chloroquine treatment. Chloroquine markedly inhibited autophagy associated pathways as shown by a reduction in autophagy pathway proteins (Atg5 and APMKβ1 Ser108), adhesion proteins (E-Cadherin, Laminin5, Integrin α5β1, FAK Tyr576/577, MMP-9), and proliferation/prosurvival proteins (BAK, C-RAF Ser338, p38 MAPK Thr180/Tyr182) (Wilcoxon p = 0.1). (blue = untreated, red = chloroquine 50 µM; n = 3, ±SEM).

Treatment of DCIS cultured cells with chloroquine phosphate (CQ, 50 µM), which disrupts autophagy, markedly suppressed xenograft tumor formation. Zero of seven DCIS cultured cell strains treated with CQ for greater than 4 days formed tumors, whereas 21 of 27 untreated cultured DCIS cell transplants yielded xenograft tumors (p<0.0003, Fisher's Exact Test). Treatment of organoids or propagated DCIS epithelial cells with CQ markedly suppressed outgrowth, spheroid formation (Figure 4), and induced apoptosis (elevation of cleaved PARP Asp214 (Figure S7)) as early as 48 hours post treatment (Figure 4). CQ treatment suppressed autophagy associated signal pathway endpoints in the DCIS progenitor cells, including IRS-1 Ser612, AKT Thr308, ERK Thr202/Tyr204, and p38 Thr180/Tyr182 (Figure S7). Statistically significant differences that provide insights into the phenotype of the spheroids before and after treatment with chloroquine treatment are shown in Figure 4E. E-cadherin, Laminin5, Integrin α5β1, FAK Tyr576/577, and MMP-9, involved in extracellular matrix signaling, attachment and degradation, were all suppressed following CQ treatment (Wilcoxon, p = 0.1). Atg5 and AMPKβ1 Ser108, components of the autophagy pathway, were suppressed, consistent with chloroquine disruption of autophagy. The remaining adherent cells following CQ treatment displayed lysosomal/autophagosome maturation arrest as evidenced by increased punctate staining with LysoTracker Red (Figure 3H). These data indicate that autophagy is required for the survival and tumorigenicity of cultured DCIS spheroid forming cells.

### Suppression of autophagy eliminates cytogenetically abnormal spheroid forming DCIS cells and suppresses their invasive phenotype

We examined the cytogenetics of CQ treated organ cultures. The surviving cells after CQ treatment were found to be cytogenetically normal (Figure S4 & S6). Thus even in an organoid culture, with a mixed cellular population, the cytogenetically abnormal spheroid forming cells, which emerged within 4 weeks, were eliminated in the culture by CQ, while the surviving cells retained the normal karyotype of the donor patient tissue. CQ treatment blocked the autophagy pathway required for the survival and 3-D growth of the cytogenetically abnormal neoplastic cells. These data indicate that autophagy is required for the survival of the spheroid forming cytogenetically abnormal DCIS cells.

The untreated DCIS neoplastic epthelial cells grew in a multilayered colony on autologous stroma, formed spheroids on the surface of the colony (Figure 4 A&B) and invaded the underlying stroma. Following treatment with CQ the epithelial colony degenerated completely. For independent patient DCIS lesions, CQ treatment administered to freshly explanted fragments of ducts prevented any outgrowth of epithelial cells for at least one month and was associated with degeneration of organoid intraductal DCIS epithelial cells. CQ treatment, administered after outgrowth had occurred for 2 weeks, markedly suppressed epithelial expansion for independent cases (Figure 4). The mean diameter of the outgrowth prior to treatment was 0.85 cm±0.11 (n = 15) and after chloroquine treatment, the mean diameter was 0.084 cm±0.03 (n = 23) (p<0.0001). In the second series of organoid cultures, the mean diameter of the outgrowth prior to treatment was 1.36±0.25 cm (n = 8) while the chloroquine treated outgrowth mean diameter was 0.21±0.03 cm (n = 7) (p = 0.0026) (Figure 4C).

CQ treatment virtually abolished spheroid and 3-D growth. As shown in Figure 4D for two different patient DCIS cases, the number of spheroids generated post chloroquine treatment was zero for the majority of explants, compared to up to 113 spheroids generated per duct organoid in the untreated culture. The number of spheroids generated in the untreated culture ranged from 1 to more than 100 for individual duct fragments (mean of 38.7±11; n = 14) (Figure 4D). Following chloroquine treatment, 12 out of 14 explants did not have any spheroids (mean number of spheroids post treatment 0.21±0.15; n = 14; p = 0.0049). CQ treatment abolished spheroid outgrowth and survival in culture. These data indicate that autophagy is necessary for the survival, growth, and invasion of the cytogenetically abnormal, tumorigenic DCIS cells.

## Discussion

Fresh human DCIS lesions reproducibly generate *in vitro* anchorage independent, neoplastic epithelial cells that generate 3-D structures including spheroids and duct-like structures. Neoplastic cells with this phenotype can emerge from a high proportion of replicate DCIS lesion samples from the same patient, and can be serially propagated for at least one year. No anchorage independent cells arose from tissue containing histologically verified normal appearing glands and adipose tissue. The anchorage independent epithelial cells were observed to arise from all grades of DCIS including ADH (Table 1).

The cytogenetically abnormal cells contained in fresh human DCIS lesions are potentially malignant by the following criteria: a) generation of neoplasms in NOD SCID mice, b) anchorage independent 3-D structures that increase in size and frequency over time, which can be subcultured for at least one year (Figure 1), c) abnormal neoplastic-type cytogenetics (Table 2, Figures S4–S6), and d) invasion of autologous stroma in the organ culture (Figure 3–4).

It is unlikely that the neoplastic cell strains propagated from fresh human DCIS ducts are isolates of micro-invasion areas or invasive cancer in the original histopathology.The patient source material was verified histologically to be devoid of microinvasion or invasive cancer before and after DCIS lesion tissue procurement. The verification was by an independent pathologist with no knowledge of the research findings for the individual specimen. The DCIS lesion source material was evaluated by IHC for type IV collagen and was found to contain an intact basement membrane (Figure S8). If the neoplastic, cytogenetically abnormal cells isolated in this study represented areas of microinvasion, this would be expected to be a rare event. This was not the case. Generation of spheroids and 3-D structures arose spontaneously from multiple, independent human DCIS duct tissue fragments from the same patient and from different patients.

The propagated spheroid forming cells, generated from fresh human DCIS tissue, were cytogenetically abnormal and were associated with xenograft tumor formation. We hypothesize that these cytogenetically abnormal cells pre-exist in the fresh human DCIS duct. This hypothesis was supported by the results of the Illumina 300,000 cytoSNP beadchip comparing the molecular cytogenetics profile of the organoid, the epithelial and cuboidal cultured monolayers, and the spheroids and 3-D structures grown in the same mixed culture. It is well established that the transition from normal to neoplastic growth involves deregulation at various cellular levels including gene-specific mutational events, alterations in signal transduction and growth control pathways, and dysfunctional DNA synthesis and repair mechanisms resulting in the generation of chromosomal abnormalities. At the chromosomal level, it is now accepted that some degree of copy number variation (CNV) and copy-neutral loss-of-heterozygosity (LOH) exists in the normal karyotype [22], [23]. However, we have observed in this study that cells with these features can rapidly emerge during the time course of *in vitro* culture. The early emergence of chromosomally abnormal cells may be the drivers of the dominant neoplastic phenotype observed during DCIS culture outgrowth.

Transformed cells containing regions of excessive CNV can change the dosage of key regulatory genes and protein output; while de novo regions of LOH can potentially expose harmful recessive alleles. Of note was the strikingly similar loss on chromosome 6 in the spheroid forming cells of three different patients. This region on chromosome 6 narrowly encompasses the SUPT3H gene (protein coding GIFtS:59, GC06M044904, UniProtKB/Swiss-Prot: SUPT3_HUMAN, O75486). Little is a known about the encoded 399 amino acid protein. It is thought to be a transcription factor, participating in the STAGA complex (SPT3-TAF9-GCN5-acetylase) involved in p53 and c-Myc regulation [24]–[26]. From this study, the genes contained within affected chromosomal regions of gain or loss can be studied in further detail to gain greater insight into the transformation process. Higher density microarrays, gene expression studies, and gene sequencing can be applied in candidate gene approaches using these data as a starting point. Even in the case in which different patients show different patterns of aberration, the same or similar regulatory pathways related to cell survival, oxidative stress, angiogenesis, or autophagy may be involved.

A strong rationale links autophagy to the survival and invasion of pre-malignant breast cancer. The first link is hypoxia and nutrient stress [27]. Proliferating ductal epithelial cells accumulating within the breast duct do not have access to the vasculature outside the duct. For this reason, high grade DCIS is associated with central necrosis, and the accumulation of lipofuschin. Autophagy is a pathway activated to promote survival in the face of hypoxic and nutrient stress [17], [28]–[30]. Consequently the activation of autophagy may divert the hypoxic cells away from apoptosis and thereby support the survival and growth of DCIS neoplastic cells within the lumen [31]. The second link is anoikis, the triggering of apoptotic cell death for cells that have been separated from their normal adhesion substratum [32]. Normal glandular epithelial cells require attachment to, or association with, the basement membrane extracellular matrix (ECM) for continued survival. During ductal hyperplasia and dysplasia epithelial cells exist within the duct at a substantial distance away from association with the peripheral basement membrane. Moreover, invading carcinoma cells can migrate into the stroma in the absence of a basement membrane anchor [33]. Autophagy has been shown to be a key regulator of survival for cells deprived of an anchoring substratum [32], and may play an important role for cell survival in any anchorage independent state. A third link is matrix degradation [29]. High grade DCIS, microinvasion, and overt carcinoma invasion are associated with interruptions, remodeling, and enzymatic breakdown of the basement membrane and the stromal ECM [34], [35]. Autophagy may facilitate cell movement through areas of degraded matrix by the phagocytic processing of matrix breakdown fragments [36]. A fourth link is calcium. Microcalcifications are mammographic indicators of high grade DCIS [37], and calcium phosphate precipitates are potent inducers of autophagy [38]. Five out of seven tissues used for ex vivo organoid culture were noted to have microcalcifications.

The data presented herein, strongly support the conclusion that autophagy plays a necessary role in the DCIS cell malignant phenotype:

a) autophagy is up-regulated in the in vivo DCIS lesion as shown by immunohistochemistry; b) autophagy is up regulated in the cultured DCIS spheroids and 3-D structures, as shown by immunohistochemistry and immunofluorescence; c) autophagy is up-regulated in mouse xenografts as shown by immunohistochemistry; d) autophagy signal proteins are up-regulated in cultured DCIS spheroids, with validation after long term culture, by reverse phase protein microarray measurement; e) disruption of autophagy by chloroquine phosphate (CQ) completely abrogated xenograft tumor formation; f) CQ completely blocked growth and invasion of DCIS cells on autologous stroma; g) CQ markedly suppressed DCIS spheroid formation and outgrowth in culture for independent experiments; and h) CQ completely eliminated cytogenetically abnormal cells from the DCIS cultured cells. Based on these established mechanistic roles, autophagy constitutes a novel target for treating DCIS and arresting DCIS transition to overt invasion.

Autophagy is an established strategy for a cell to avoid apoptosis in the face of oxidative, hypoxic and nutrient deprivation stress. Our results indicate that autophagy is a requirement for the survival and the malignant phenotype (tumorigenicity and invasion) of cytogenetically abnormal cultured human DCIS cells. It is possible that the observed genetic abnormalities directly or indirectly induced the up-regulation of autophagy and thereby promoted survival of the DCIS cells. It is also possible that autophagy was not driven directly by the genetic changes. Instead the malignant precursor cells up-regulated autophagy as a necessary means to generate anchorage independent 3-D structures such as spheroids and pseudo ducts. In either case, the end result is the same: Autophagy is required for the observed phenotype.

Chloroquine phosphate, which suppressed or abolished the anchorage independent, cytogenetically abnormal anchorage independent neoplastic DCIS cells, is an orally administered small molecule inhibitor which blocks the autophagy pathway by accumulating in autophagosomes and inhibiting autophagosomal formation/function [17]. Chloroquine has been shown to suppress N-methyl-N-nitrosurea induced mouse breast carcinomagenesis [39], enhances the effectiveness of tyrosine kinase inhibitor treatment of primary CML stem cells [40], and has been proposed as a potential means to enhance the effectiveness of tamoxifen *in vitro* in tamoxifen resistant breast carcinoma cells by blocking autophagy dependent cell survival [17], [28]. Moreover, chloroquine has been proposed as a chemoprevention therapy for Myc induced lymphomagenesis because it induces lysosomal stress that causes a p53 dependent cell death that does not require caspase mediated apoptosis [41].

These data provide additional evidence that the local ductal tissue microenvironment influences the progression of in situ carcinomas. Breast stromal cells [42], endothelial cells, and myoepithelial cells [43] have been implicated in the transition from in situ to invasive carcinoma. By IHC, Caveolin-1 was found to be down regulated in stroma of DCIS lesions with a greater incidence of subsequent invasive cancer [44]. Jedeszko *et al* [34] reported that stromal fibroblasts contributed to the regulation of invasion by the production of hepatocyte growth factor, using a co-culture model with MCF10 and SUM102 cell lines. The present study identifies and characterizes malignant precursor epithelial cells in fresh human DCIS lesions, and therefore goes beyond model systems using established cell lines such as MCF10.

Even though the cytogenetically abnormal DCIS epithelial cells we have derived appear to have the characteristics of progenitor cells by RPMA analysis (Figure 2 and Table S2), our method of generating these cells is different than cell sorting methods used for what are defined as “cancer stem cells”. In the present study we purposely did not assume that the DCIS neoplastic cells would be positive for CD44 or any other surface marker. We did not enzymatically dissociate the DCIS lesions and then conduct flow sorting as has been done for studies of breast carcinoma [45]–[47]. Instead, we allowed the spheroid and 3-D ductal structures to grow spontaneously from epithelial cells that emerged from exposed open ends of the DCIS duct in organ culture. Thus, the cytogenetically abnormal (Table 2) progenitor-type cells we have identified are self-selected by anchorage independence in organ culture. While there is broad evidence supporting the existence and role of stem cells both in normal mammary gland development and tumorigenesis [45]–[48], we cannot formally classify these cells to be, or not to be, “cancer stem cells”. This classification is irrelevant to the fact that these propagated DCIS lesion derived cells are cytogenetically abnormal and have malignant characteristics.

In conclusion, fresh human DCIS organoid culture reveals cytogenetically abnormal, neoplastic, anchorage independent cells that require autophagy for survival. Invasive neoplastic cells may pre-exist within the human breast DCIS duct but are apparently held in check by the ductal niche, and can be coaxed to emerge in organ culture when the duct is cut open. Blockade of the autophagy pathway abolishes the propagation, invasion, and 3-D growth of the DCIS neoplastic cells. The reproducible derivation of cytogenetically abnormal cells with fresh human DCIS lesions provides a new *ex vivo* model to study the biology of DCIS. Moreover, the yield, and properties, of progenitor-like cells from samples of human DCIS lesions following *in vivo* treatment of DCIS can be used to screen the effectiveness of an *in vivo* therapy. Finally, autophagy constitutes a new treatment target for DCIS.

## Materials and Methods

### Ethics Statement

This study was conducted according to the principles expressed in the Declaration of Helsinki, with written informed patient consent. The study was approved by the institutional review boards of Inova Fairfax Hospital and George Mason University. Animal care and use was approved by Institutional Animal Care and Use Committee of George Mason University and the US Department of Defense according to (a) DOD Directive 3216.1, “The Use of Laboratory Animals in DOD Programs”, (b) US Army Regulation 40–33, “The Care and Use of Laboratory Animals in DOD Programs”, and (c) Animal Welfare Regulations (CFR Title 9, Chapter 1, Subchapter A, Parts 1–3).

### Tissue collection

Fresh, sterile breast DCIS tissue was obtained from patients undergoing standard of care surgery for suspected or biopsy confirmed neoplasia at Inova Fairfax Hospital, Falls Church, VA (Table 1). Written informed consent was obtained prior to tissue procurement according to the provisions of the Declaration of Helsinki and Inova Health System institutional review board. Gross tissue pathology at the time of procurement was assessed by a board certified pathologist (LP, SZ, GM, JM, JJ). Tissue containing DCIS lesions was excised for further macrodissection. Ductal tissue was dissected from surrounding breast adipose/fibrous tissue of the surgical specimen. The ductal tissue was rinsed in serum free DMEM/F12 medium (Invitrogen, Carlsbad, CA) prior to distribution in culture flasks.

### Organoid culture of fresh human DCIS lesions

The tissue was dissected into organoids approximately 3 mm^2^, containing one or more discernable duct segments with associated stroma. Dissected breast ductal tissue was grown in serum free DMEM/F12 medium supplemented with human recombinant EGF (10 ng/mL; Cell Signaling Technology, Danvers, MA), insulin (10 µg/mL; Roche, Indianapolis, IN), streptomycin sulfate (100 µg/mL; Sigma, St. Louis, MO) and gentamicin sulfate (20 µg/mL; Sigma), with or without 0.36% (v/v) murine Engelbreth-Holm-Swarm (EHS) derived, growth factor reduced, basement membrane extract (Trevigen, Gaithersburg, MD) at 37°C in a humidified 5.0% CO_2_ atmosphere. Medium was replaced three times per week. Organoids were submerged in a minimum volume of medium (just enough to cover the duct fragments) to maximize gas exchange. Non-adherent organoids were removed from the culture flask. Cultures were maintained continuously for up to one year. Periodically, organoids were removed, under microscopic visualization, for propagation into new culture flasks or phenotypic and molecular analysis.

### Pharmacological inhibition of autophagy

Autophagy was inhibited in organoid cultures by treating cultures with chloroquine diphosphate (CQ) (50 µM; Sigma) in DMEM/F12 medium as described above. CQ-containing medium was replaced three times per week for a period of 6 months. Comparable untreated control cultures were maintained in identical medium lacking chloroquine with similar media changes.

### Murine xenograft generation

Human DCIS tumor tissue, propagated organoid tissue, or spheroids generated from primary DCIS organoid cultures were transplanted transdermally into the mammary fat pad of female NOD/SCID mice (NOD.CB17-*Prkdc* ^scid^/NCrHsd; Harlan or The Jackson Laboratory) with a 17β Estradiol pellet (Innovative Research of America, Sarasota, FL; 60 day release, 1.7 mg/pellet). The DCIS organoids, prior to transplantation into the murine mammary fat pad, were incubated in DMEM/F12 medium supplemented with EGF, insulin, streptomycin, gentamicin, or estrogen (Sigma) for 6 to 18 hours. The study was conducted under a contract with Biocon Inc., Rockville, MD.

Survival, weight, and condition of all mice were monitored daily and palpable tumor masses were measured with calipers (length/width) twice weekly. Mice exhibiting evidence of tumor growth were sacrificed as necessary or after 120 days. Complete necropsies were performed for evidence of metastasis. Tumor tissue, when present, was either excised for immunohistochemical analysis or injected into a different NOD SCID mouse for propagation of the tumor.

### Immunohistochemistry

Formalin fixed murine tissue or DCIS organoids were processed and paraffin embedded by commercial laboratories (AML Laboratories, Inc, Rosedale, MD or Bi-Biomics, Nampa, ID). Formalin fixed paraffin embedded (FFPE) tissue sections (5 µm or 1 µm thickness) mounted on positively charged glass slides were baked at 56°C for 20 min., deparaffinized in xylene and rehydrated in a series of graded alcohols (100%, 95%, and 70%) with a final rinse in wash buffer (Dako, Carpinteria, CA). Immunostaining was performed on a Dako Autostainer with an Envision+HRP staining kit (Dako) per manufacturer's instructions (Table S3). Stained tissue sections were counterstained with Hematoxylin (Dako). Images were captured with an Olympus BX51 microscope using 10×, 20×, or 100× objectives.

### Immunofluorescence and confocal imaging

Spheroids were aspirated directly from the culture flask under direct microscopic visualization, mounted on positively charged glass microscope slides, fixed in 16% paraformaldehyde (Fisher Scientific), and stored dessicated at 4°C. FFPE murine xenograft tissue sections were deparaffinized in xylene, and rehydrated in graded alcohols. Spheroids and FFPE sections were incubated at room temperature with anti-human specific epithelial antigen conjugated to FITC (EpCAM-FITC, 5 µg/mL) (Abcam, Cambridge, MA), or mouse immunoglobulin IgG1 as a negative control (Dako). Slides were rinsed in borate buffer pH 8, then nuclear counterstained with ProLong Gold+DAPI (Invitrogen). Images were captured with a Nikon Eclipse C1si confocal microscope in different channels for EpCAM-FITC (pseudo-colored green, 488 nm) and DAPI (psuedo-colored blue, 408 nm) using the 20× objective.

### Autophagosome/lysosome imaging

LysoTracker Red (Invitrogen; 75 nM) and nuclear counterstain Hoechst 33258 pentahydrate (Invitrogen; 5 µg/mL) were added to DMEM/F12 culture medium as described above and incubated with live human DCIS organoid cell cultures for 0.5 hour. Medium containing dye was removed and replaced with fresh medium. Images were captured with either a Nikon Eclipse C1si confocal or a Nikon Eclipse TE200 microscope in different channels for LysoTracker Red (psuedo-colored red, 561 nm) and Hoechst 33258 (pseudo-colored blue) using either the 10× or 20× objective.

### Cell signaling pathway mappping by reverse phase protein microarray (RPMA)

Cellular outgrowths were removed from the culture flask by scraping or aspiration with a pipette and were spun briefly to pellet the cells. Medium was removed by aspiration and the cell pellet was subjected to lysis with a 10% (v/v) solution of Tris(2-carboxyethyl)phosphine (TCEP; Pierce, Rockford, IL) in Tissue Protein Extraction Reagent (T-PER™, Pierce)/Tris-glycine 2X SDS buffer (Invitrogen). Cell lysates were stored at –80°C prior to microarray construction. Cellular lysates were printed on glass backed nitrocellulose array slides (FAST Slides, Whatman, Florham Park, NJ) using an Aushon 2470 arrayer (Aushon BioSystems, Burlington, MA) equipped with 350 µm pins as previously described [49]. Immunostaining was performed as previously described on a Dako Autostainer per manufacturer's instructions (CSA kit, Dako) [49]. Each slide was incubated with a single primary antibody at room temperature for 30 minutes. Polyclonal and monoclonal antibodies are listed in Table S1. Antibodies were validated by western blotting as previously described [19]. The negative control slide was incubated with antibody diluent. Secondary antibody was goat anti-rabbit IgG H+L (1∶7,500) (Vector Labs, Burlingame, CA) or rabbit anti-mouse IgG (1∶10) (Dako). Subsequent signal detection was amplified via horseradish peroxidase mediated biotinyl tyramide deposition with chromogenic detection (Diaminobenzidine) per manufacturer's instructions (Dako).

Total protein per microarray spot was determined with a Sypro Ruby protein stain (Invitrogen/Molecular Probes) per manufacturer's directions and imaged with a CCD camera (NovaRay, Alpha Innotech, San Leandro, CA).

### Molecular Cytogenetics

Cellular outgrowths were removed from the culture flask by scraping or aspiration with a pipette and were spun briefly to pellet the cells. Culture medium was removed by aspiration and the cell pellet was immediately frozen on dry ice and stored at –80°C prior to nucleic acid extraction. Nucleic acid preparations derived from human breast tissue and/or cell culture out growths were tested using quantitative PCR (qPCR), PicoGreen (Invitrogen) staining and fluorometry (FLx800 fluorescence plate reader, BioTek, Winooski, VT). Microarray-based genomic analysis was performed using CytoSNP-12 beadchips (Illumina, Inc.) and analyzed on an Illumina BeadStation 500 GX laser scanner [50]–[52]. Briefly, the microarray process involved sample DNA amplification, followed by DNA fragmentation, hybridization of samples to beadchips, single-nucleotide extension, antibody-based labeling, and finally two-color fluorescence scanning and computer-based raw data collection.

The DNA extraction and purification was performed using a DNA purification column (QIAmp DNA Mini Kit, Qiagen, Valencia, CA). Approximately 200 ng of DNA at a concentration of 50 ng/µL was amplified, fragmented, precipitated, re-suspended, and hybridized to the Illumina CytoSNP-12 beadchips. After single-base extension, sample DNA was stained and the chip was washed, dried, and scanned for the resulting 300,000 SNP calls and copy number values.

Raw fluorescence data was converted to genotypic data using the Illumina GenomeStudio software program. Data analysis was performed using the Illumina KaryoStudio software program that converts genotypic and signal intensity data into a “molecular karyotype” showing B allele frequency, Log R ratio, LOH score and Copy Number Score. Log R ratio, which is the log (base 2) ratio of the normalized R value for the particular SNP divided by the expected normalized R value, was employed. The red line in the log R plot indicates a smoothing series with a 200 kb moving average window. Thus, a Log R Ratio\2 was considered to represent a true amplification and Log R Ratio\-1.5 was considered to represent a probable homozygous deletion. Additionally, B allele frequency data was used to identify regions of copy-neutral and hemizygous LOH.

### Statistics

Standard deviation (SD) or standard error of the mean (SEM) was calculated for small group comparisons. The Student t-test, two tailed with Welch's correction, was used to calculate the p-value, and to determine the statistical difference of epithelial outgrowth area before and after CQ treatment and spheroid generation. P values <0.05 were considered significant (GraphPad Prism ver 5.03, GraphPad Software). Wilcoxon rank sum was used to determine the differences between CQ treated and untreated groups for the reverse phase protein arrays (R, SAS Institute). A p = 0.1 was considered different for small sample sizes.

## Supporting Information

Figure S1 Macroautophagy cell signaling pathway. Autophagy (auto - self, phagy - eating) is a catabolic process that can either maintain cellular homeostasis or result in cell death. Intracellular signaling kinases such as AKT, PI3 Kinase, ERK, Bcl-2, and mTOR regulate autophagy. Reverse phase protein microarrays (RPMA) were employed in the present study to evaluate the activation (phosphorylation) of signal pathway proteins that are associated with autophagy. Pathway diagram reproduced courtesy of Cell Signaling Technology, Inc. (www.cellsignal.com).
(0.93 MB TIF)

Figure S2 Human DCIS xenograft tumor histology. H&E staining of mouse xenograft tumor derived from fresh human DCIS cells. (A) Human DCIS explant derived mouse tumor (20x). (B) Xenograft tumor is comprised of pleomorphic epithelial cells with prominent nucleoli and partial ductal/glandular differentiation directly abutting or invading surrounding stroma (40x).
(7.27 MB TIF)

Figure S3 Molecular karyotype of chromosome 6 from human cultured DCIS cells shows a deletion p21.1/12.3. The upper panels show the log R ratio plots from 3 different patients (top: 09-148 spheroids/3-D structure; middle: 08-352 3-D structure; bottom: 09-091 spheroids/3-D structure). These data represent DNA ploidy, or copy number, for the displayed chromosomal region with the red line indicating the statistical average value. A log R ratio of 0.0 equals a DNA copy number of 2 (diploid). Deflection downward of the red line indicates loss of DNA copy number. Each blue dot represents the log R ratio value for each SNP. The shaded regions represent segments of DNA deviating from a copy number of 2 as determined by the Illumina GenomeStudio 2.0 software. The software uses both quantitative fluorescence intensity and qualitative genotypic data for determining copy number values. The color code is as follows: orange indicates a region of 1 copy; red, 0 copies; blue, 3 copies; purple, 4 or more copies; and green, copy-neutral LOH (2 copies). The center panel shows the chromosomal ideogram indicating cytological bands with the centromere in red. The small window shows the region expanded in the figure and the nucleotide positions for this region are shown below the ideogram. The lower panel shows the cytogenetic bands and genetic map for genes located in the expanded region. Note that the region of the deletion for these 3 patients (orange) corresponds to the transcript for SUPT3H.
(0.18 MB TIF)

Figure S4 Molecular karyotype of chromosome 5 from chloroquine treated or untreated cultured human DCIS cells. The upper panel shows log 2 ratio plots of 2 different samples from the same patient (top: 09-148 chloroquine treated epithelial monolayer; bottom: 09-148 untreated spheroids/3-D structure). In the upper panel, the top plot shows the log R ratio from chloroquine treated human DCIS cell cultures showing normal ploidy, while the lower plot shows a number of extended regions of gain and loss of content on chromosome 5. The color code is as follows: orange indicates a region of 1 copy; red, 0 copies; blue, 3 copies; purple, 4 or more copies; and green, copy-neutral LOH (2 copies). Blue and purple regions show an increase of copy number extending from nucleotide position ∼31 Mb to ∼43 Mb (12 Mb in total) affecting the dosage of numerous genes. Additional regions of copy number gain are present distally, including subtelomeric regions. Extended regions of copy number loss are indicated in orange (one copy) and red (0 copy). The lower panel shows the cytogenetic banding pattern and the corresponding nucleotide positions beginning with the p-telomere.
(0.22 MB TIF)

Figure S5 Allele frequency of chromosome 17 q-arm for spheroids from DCIS organoid culture. Molecular karyotype of chromosome 17 q arm. The upper panel shows log R ratio and B allele frequency plots of genomic DNA from an organoid sample (case 09-148 spheroids/3-D structure). The top plot shows an upper deflection of the red averaging line indicating a gain in copy number (blue) spanning ∼14 Mb of chromosomal content. This is the largest of multiple gain of copy number regions on the q-arm in this neoplastic sample (data not shown). The bottom plot shows the B allele frequency data for this same region. Within the blue shaded region, the B allele frequency data for heterogeneous SNPs (normally at 0.5 for diploid) is split into 2 lines at values above and below 0.5, indicating the presence of 3 copies of DNA in this region, consistent with the log 2 ratio data shown above. The bottom panel shows the chromosome 17 ideogram with the expanded region outlined below. The gain of DNA copy number extends from q22 to q24.3.
(0.15 MB TIF)

Figure S6 Molecular karyotype of chromosome 6 from chloroquine treated or untreated cultured human DCIS cells. Molecular karyotype of chromosome 6 from chloroquine treated cultured DCIS epithelial monolayer devoid of spheroids and untreated spheroids for case 09-148. Molecular karyotype of chromosome 6; p21.1/p12.3: The upper panel shows the log R ratio plots from 2 different samples from the same patient. In the upper panel, the top plot shows the log R ratio from cell cultures treated with chloroquine phosphate showing normal ploidy, while the lower plot shows a hemizygous deletion (orange) of the SUPT3H locus in cultures displaying spheroid and 3-D structures. This data represents DNA ploidy, or copy number, for the displayed chromosomal region with the red line indicating the statistical average value. A log R ratio of 0.0 equals a DNA copy number of 2 (diploid). Deflection downward of the red line indicates loss of DNA copy number. Each blue dot represents the log R ratio value for each SNP. The shaded regions represent segments of DNA deviating from a copy number of 2 as determined by the Illumina GenomeStudio 2.0 software. The software uses both quantitative fluorescence intensity and qualitative genotypic data for determining copy number values. The color code is as follows: orange indicates a region of 1 copy; red, 0 copies; blue, 3 copies; purple, 4 or more copies; and green, copy-neutral LOH (2 copies). The center panel shows the chromosomal ideogram indicating cytological bands with the centromere in red. The small window shows the region expanded in the figure and the nucleotide positions for this region are shown below the ideogram. The lower panel shows the cytogenetic bands and genetic map for genes located in the expanded region.
(0.58 MB TIF)

Figure S7 Signal pathway alterations induced by chloroquine treatment of cultured DCIS spheroids. Complete set of cell signaling kinases measured by reverse phase protein microarray for the subset shown in Figure 4. Chloroquine markedly inhibited autophagy associated pathways as shown by a reduction in autophagy pathway proteins (Atg5 and APMKβ1 Ser108), adhesion proteins (E-Cadherin, Laminin5, Integrin α5β1, FAK Tyr576/577, MMP-9), and proliferation/prosurvival proteins (BAK, C-RAF Ser338, p38 MAPK Thr180/Tyr182) (blue bar - untreated, red bar - chloroquine treated (50 µM), n = 3, ±SEM; Wilcoxon p = 0.1).
(0.46 MB TIF)

Figure S8 Basement membrane remains intact in DCIS cultured organoids. Collagen type IV immunohistochemistry demonstrates intact basement membrane surrounding breast ducts (brown staining). (A) Case 08-352 FFPE primary breast tissue at time of procurement. Scale bar 100 µm, magnification 10×. (B) The basement membrane remains intact surrounding the duct that is within the tissue fragment and surviving after the breast organoid was growing in culture for 12 weeks (case 08-148, magnification 20×).
(2.39 MB TIF)

Table S1 Validated primary antibodies for Reverse Phase Protein Microarray analysis.
(0.03 MB XLS)

Table S2 Verification of DCIS progenitor cell phenotype: Reverse Phase Protein Microarray analysis of DCIS cultured epithelial cells, spheroids and stromal fibroblasts.
(0.04 MB XLS)

Table S3 Primary antibodies and antigen retrieval conditions for immunohistochemistry.
(0.03 MB DOC)
