# Supplementary figures and images for: Malignant Precursor Cells Pre-Exist in Human Breast DCIS and Require Autophagy for Survival
Source: PLoS One. 2010 Apr 20;5(4):e10240. doi: 10.1371/journal.pone.0010240 (PMC2857649; doi:10.1371/journal.pone.0010240)

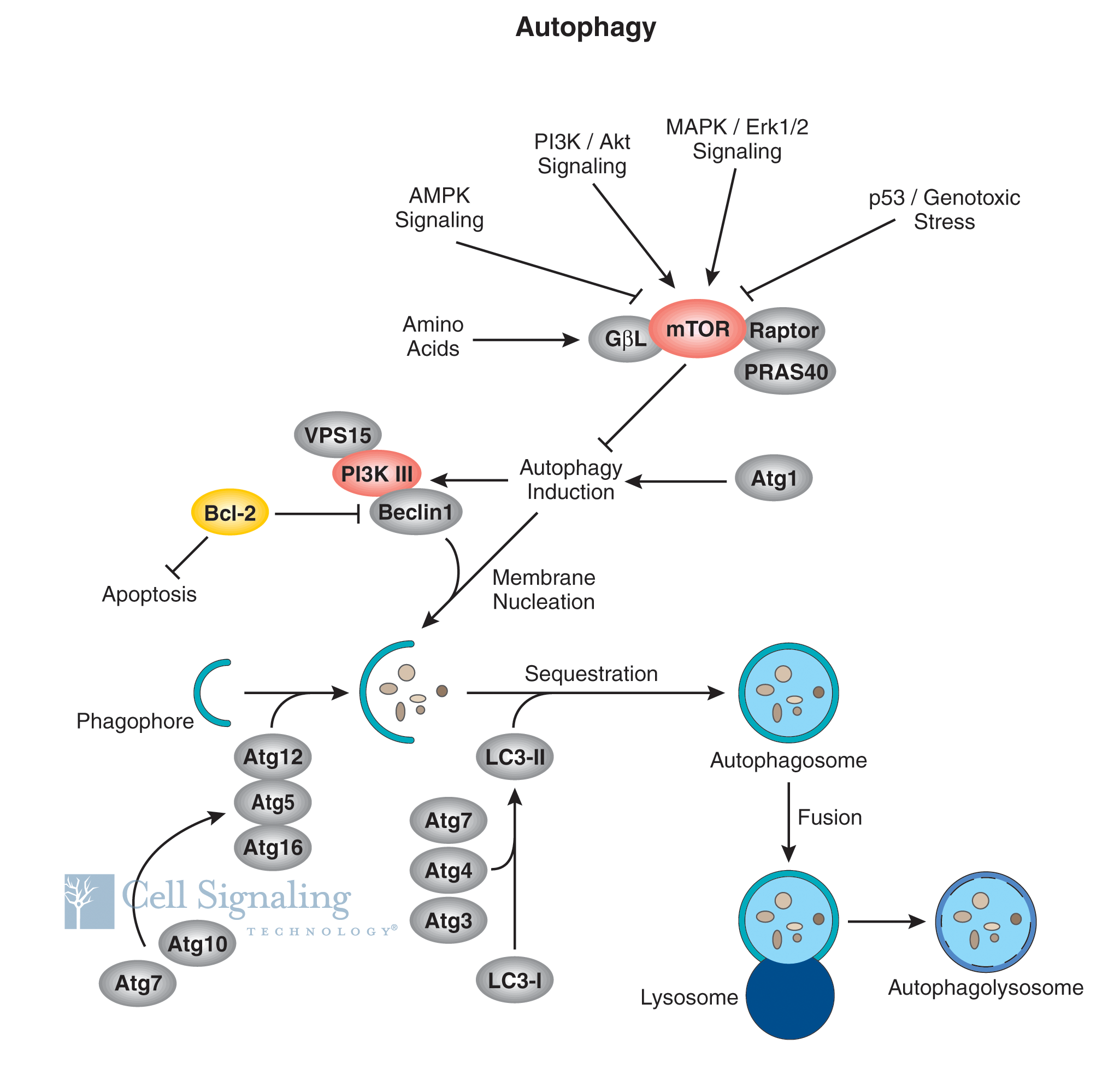

Supplement: Figure S1 — Macroautophagy cell signaling pathway. Autophagy (auto - self, phagy - eating) is a catabolic process that can either maintain cellular homeostasis or result in cell death. Intracellular signaling kinases such as AKT, PI3 Kinase, ERK, Bcl-2, and mTOR regulate autophagy. Reverse phase protein microarrays (RPMA) were employed in the present study to evaluate the activation (phosphorylation) of signal pathway proteins that are associated with autophagy. Pathway diagram reproduced courtesy of Cell Signaling Technology, Inc. (www.cellsignal.com). (0.93 MB TIF) [file pone.0010240.s001.tif]

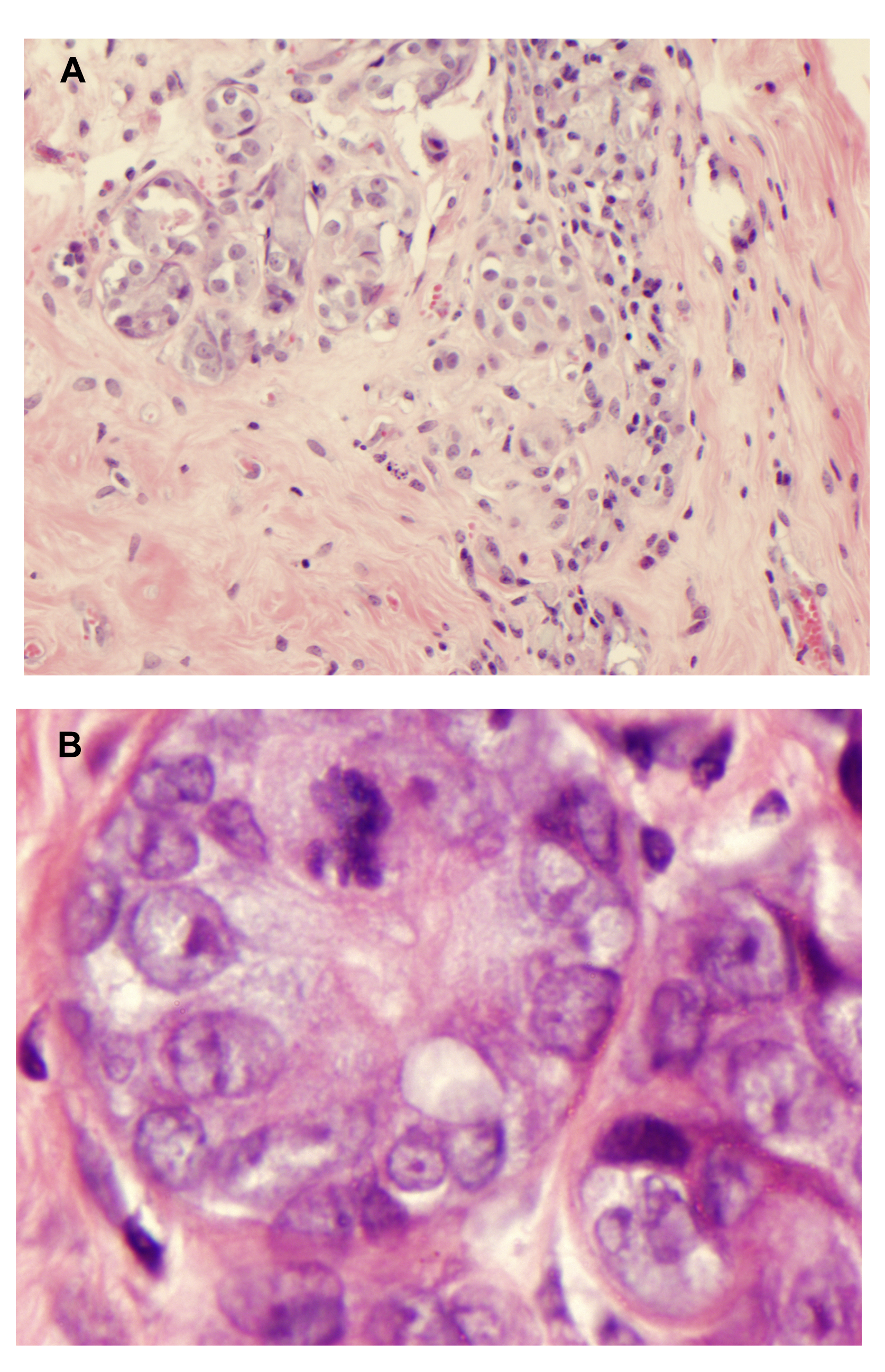

Supplement: Figure S2 — Human DCIS xenograft tumor histology. H&E staining of mouse xenograft tumor derived from fresh human DCIS cells. (A) Human DCIS explant derived mouse tumor (20x). (B) Xenograft tumor is comprised of pleomorphic epithelial cells with prominent nucleoli and partial ductal/glandular differentiation directly abutting or invading surrounding stroma (40x). (7.27 MB TIF) [file pone.0010240.s002.tif]

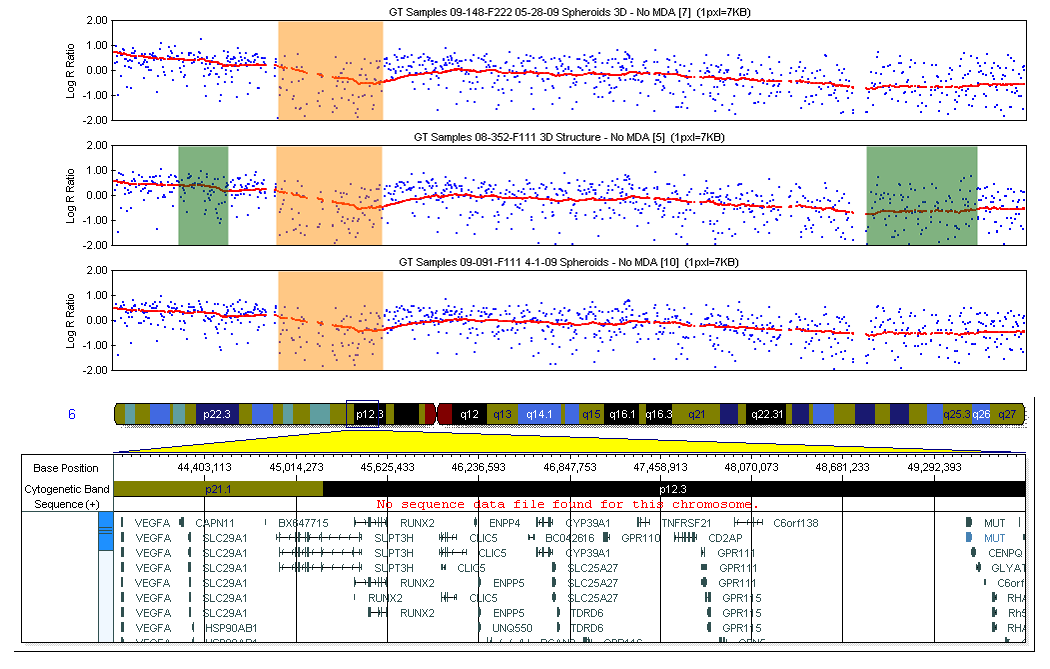

Supplement: Figure S3 — Molecular karyotype of chromosome 6 from human cultured DCIS cells shows a deletion p21.1/12.3. The upper panels show the log R ratio plots from 3 different patients (top: 09-148 spheroids/3-D structure; middle: 08-352 3-D structure; bottom: 09-091 spheroids/3-D structure). These data represent DNA ploidy, or copy number, for the displayed chromosomal region with the red line indicating the statistical average value. A log R ratio of 0.0 equals a DNA copy number of 2 (diploid). Deflection downward of the red line indicates loss of DNA copy number. Each blue dot represents the log R ratio value for each SNP. The shaded regions represent segments of DNA deviating from a copy number of 2 as determined by the Illumina GenomeStudio 2.0 software. The software uses both quantitative fluorescence intensity and qualitative genotypic data for determining copy number values. The color code is as follows: orange indicates a region of 1 copy; red, 0 copies; blue, 3 copies; purple, 4 or more copies; and green, copy-neutral LOH (2 copies). The center panel shows the chromosomal ideogram indicating cytological bands with the centromere in red. The small window shows the region expanded in the figure and the nucleotide positions for this region are shown below the ideogram. The lower panel shows the cytogenetic bands and genetic map for genes located in the expanded region. Note that the region of the deletion for these 3 patients (orange) corresponds to the transcript for SUPT3H. (0.18 MB TIF) [file pone.0010240.s003.tif]

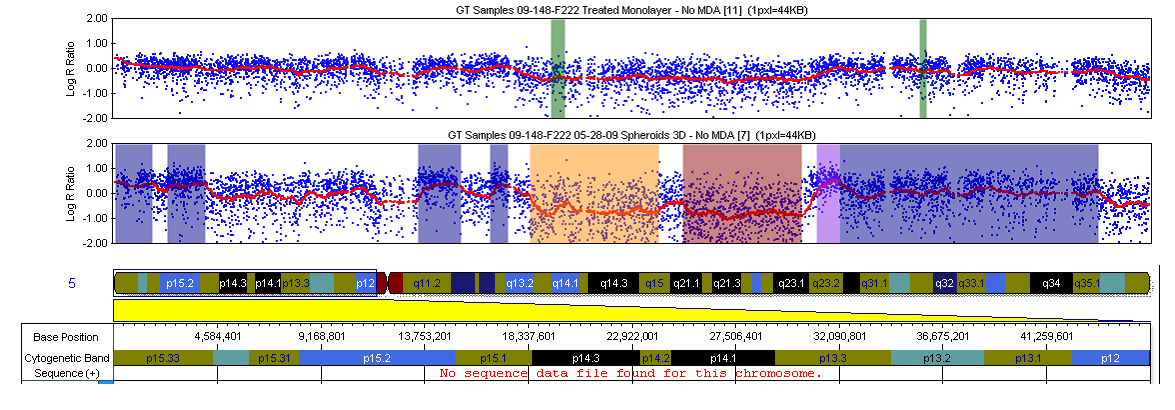

Supplement: Figure S4 — Molecular karyotype of chromosome 5 from chloroquine treated or untreated cultured human DCIS cells. The upper panel shows log 2 ratio plots of 2 different samples from the same patient (top: 09-148 chloroquine treated epithelial monolayer; bottom: 09-148 untreated spheroids/3-D structure). In the upper panel, the top plot shows the log R ratio from chloroquine treated human DCIS cell cultures showing normal ploidy, while the lower plot shows a number of extended regions of gain and loss of content on chromosome 5. The color code is as follows: orange indicates a region of 1 copy; red, 0 copies; blue, 3 copies; purple, 4 or more copies; and green, copy-neutral LOH (2 copies). Blue and purple regions show an increase of copy number extending from nucleotide position ∼31 Mb to ∼43 Mb (12 Mb in total) affecting the dosage of numerous genes. Additional regions of copy number gain are present distally, including subtelomeric regions. Extended regions of copy number loss are indicated in orange (one copy) and red (0 copy). The lower panel shows the cytogenetic banding pattern and the corresponding nucleotide positions beginning with the p-telomere. (0.22 MB TIF) [file pone.0010240.s004.tif]

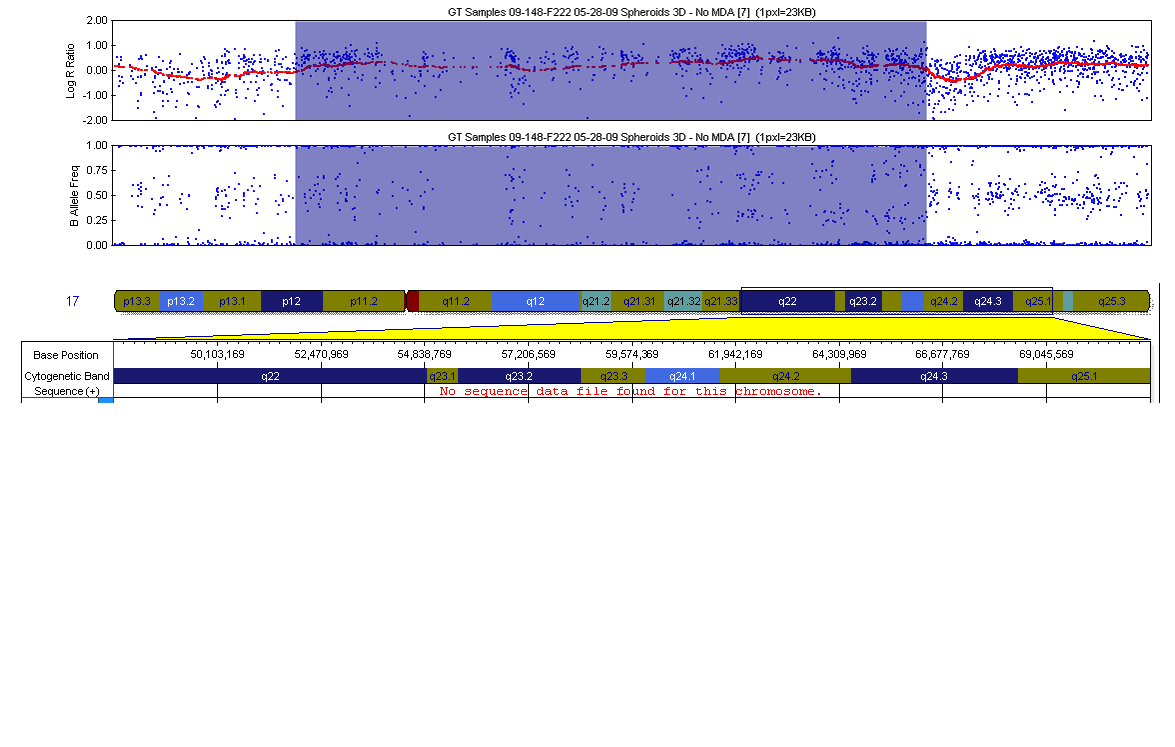

Supplement: Figure S5 — Allele frequency of chromosome 17 q-arm for spheroids from DCIS organoid culture. Molecular karyotype of chromosome 17 q arm. The upper panel shows log R ratio and B allele frequency plots of genomic DNA from an organoid sample (case 09-148 spheroids/3-D structure). The top plot shows an upper deflection of the red averaging line indicating a gain in copy number (blue) spanning ∼14 Mb of chromosomal content. This is the largest of multiple gain of copy number regions on the q-arm in this neoplastic sample (data not shown). The bottom plot shows the B allele frequency data for this same region. Within the blue shaded region, the B allele frequency data for heterogeneous SNPs (normally at 0.5 for diploid) is split into 2 lines at values above and below 0.5, indicating the presence of 3 copies of DNA in this region, consistent with the log 2 ratio data shown above. The bottom panel shows the chromosome 17 ideogram with the expanded region outlined below. The gain of DNA copy number extends from q22 to q24.3. (0.15 MB TIF) [file pone.0010240.s005.tif]

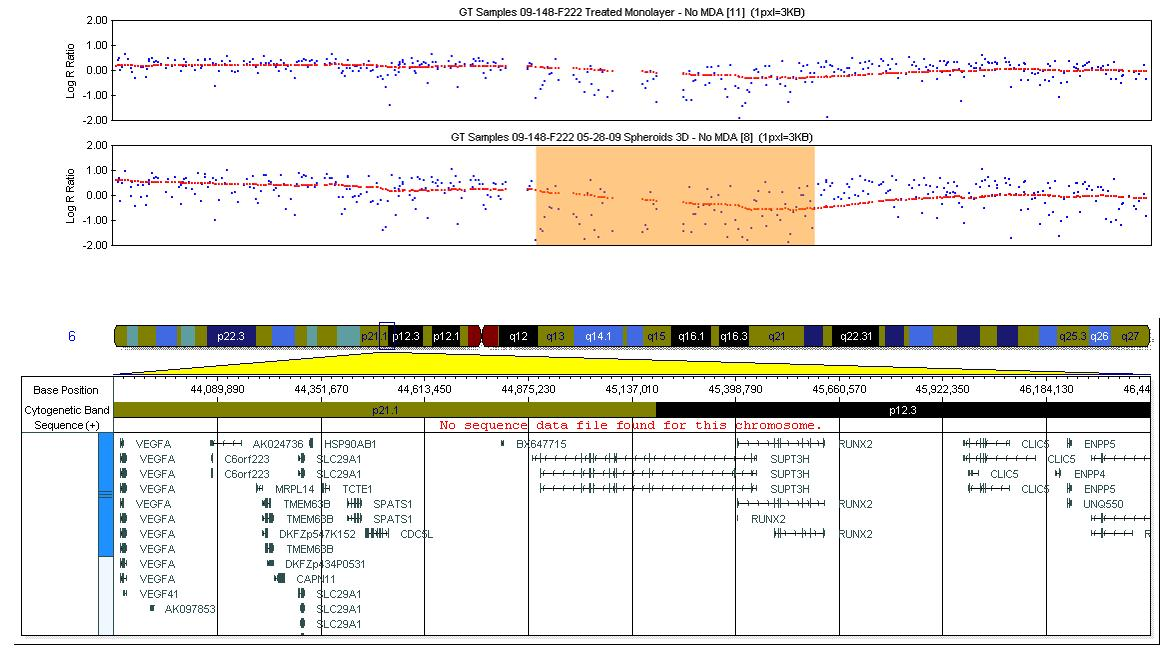

Supplement: Figure S6 — Molecular karyotype of chromosome 6 from chloroquine treated or untreated cultured human DCIS cells. Molecular karyotype of chromosome 6 from chloroquine treated cultured DCIS epithelial monolayer devoid of spheroids and untreated spheroids for case 09-148. Molecular karyotype of chromosome 6; p21.1/p12.3: The upper panel shows the log R ratio plots from 2 different samples from the same patient. In the upper panel, the top plot shows the log R ratio from cell cultures treated with chloroquine phosphate showing normal ploidy, while the lower plot shows a hemizygous deletion (orange) of the SUPT3H locus in cultures displaying spheroid and 3-D structures. This data represents DNA ploidy, or copy number, for the displayed chromosomal region with the red line indicating the statistical average value. A log R ratio of 0.0 equals a DNA copy number of 2 (diploid). Deflection downward of the red line indicates loss of DNA copy number. Each blue dot represents the log R ratio value for each SNP. The shaded regions represent segments of DNA deviating from a copy number of 2 as determined by the Illumina GenomeStudio 2.0 software. The software uses both quantitative fluorescence intensity and qualitative genotypic data for determining copy number values. The color code is as follows: orange indicates a region of 1 copy; red, 0 copies; blue, 3 copies; purple, 4 or more copies; and green, copy-neutral LOH (2 copies). The center panel shows the chromosomal ideogram indicating cytological bands with the centromere in red. The small window shows the region expanded in the figure and the nucleotide positions for this region are shown below the ideogram. The lower panel shows the cytogenetic bands and genetic map for genes located in the expanded region. (0.58 MB TIF) [file pone.0010240.s006.tif]

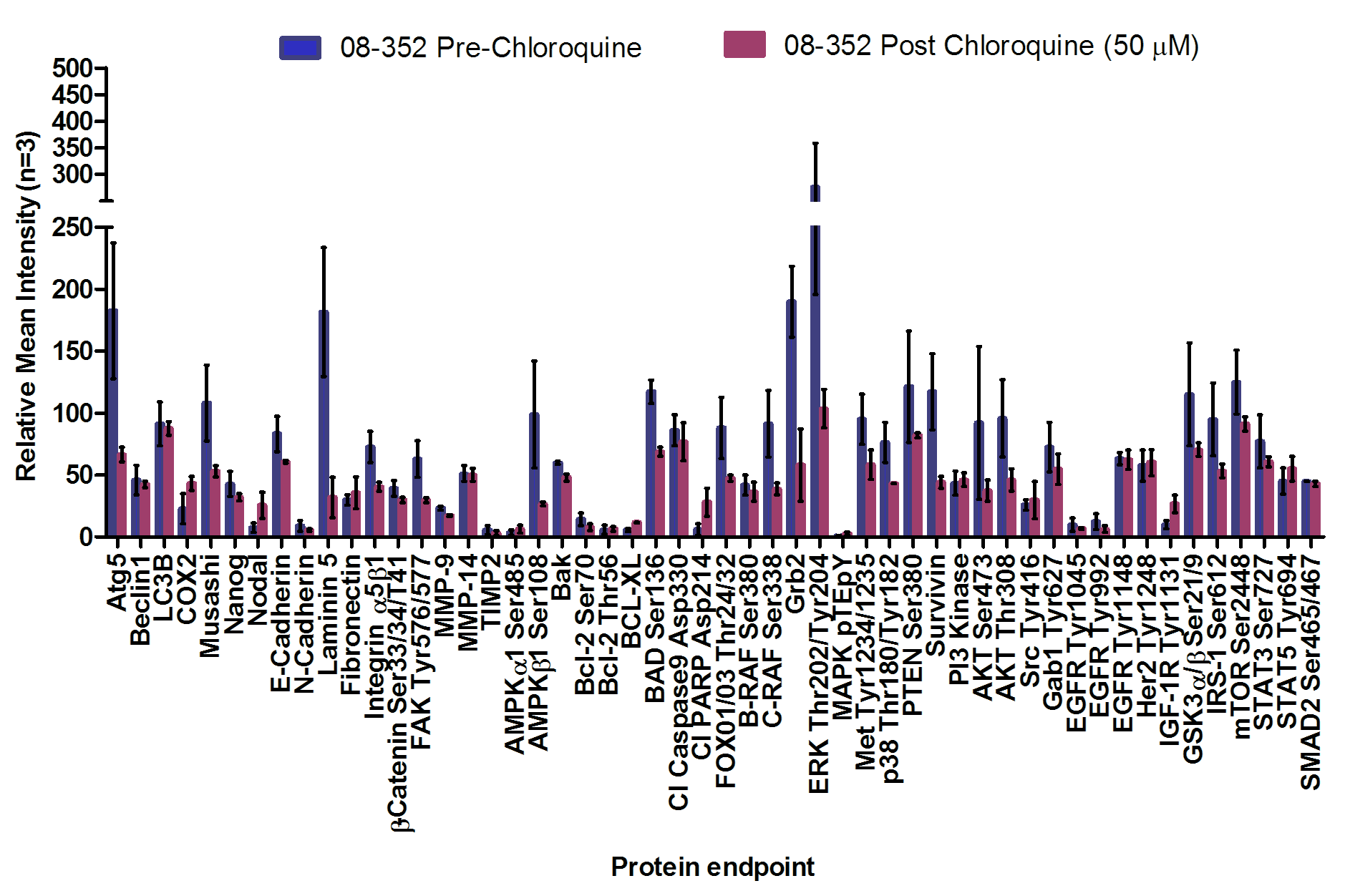

Supplement: Figure S7 — Signal pathway alterations induced by chloroquine treatment of cultured DCIS spheroids. Complete set of cell signaling kinases measured by reverse phase protein microarray for the subset shown in Figure 4. Chloroquine markedly inhibited autophagy associated pathways as shown by a reduction in autophagy pathway proteins (Atg5 and APMKβ1 Ser108), adhesion proteins (E-Cadherin, Laminin5, Integrin α5β1, FAK Tyr576/577, MMP-9), and proliferation/prosurvival proteins (BAK, C-RAF Ser338, p38 MAPK Thr180/Tyr182) (blue bar - untreated, red bar - chloroquine treated (50 µM), n = 3, ±SEM; Wilcoxon p = 0.1). (0.46 MB TIF) [file pone.0010240.s007.tif]

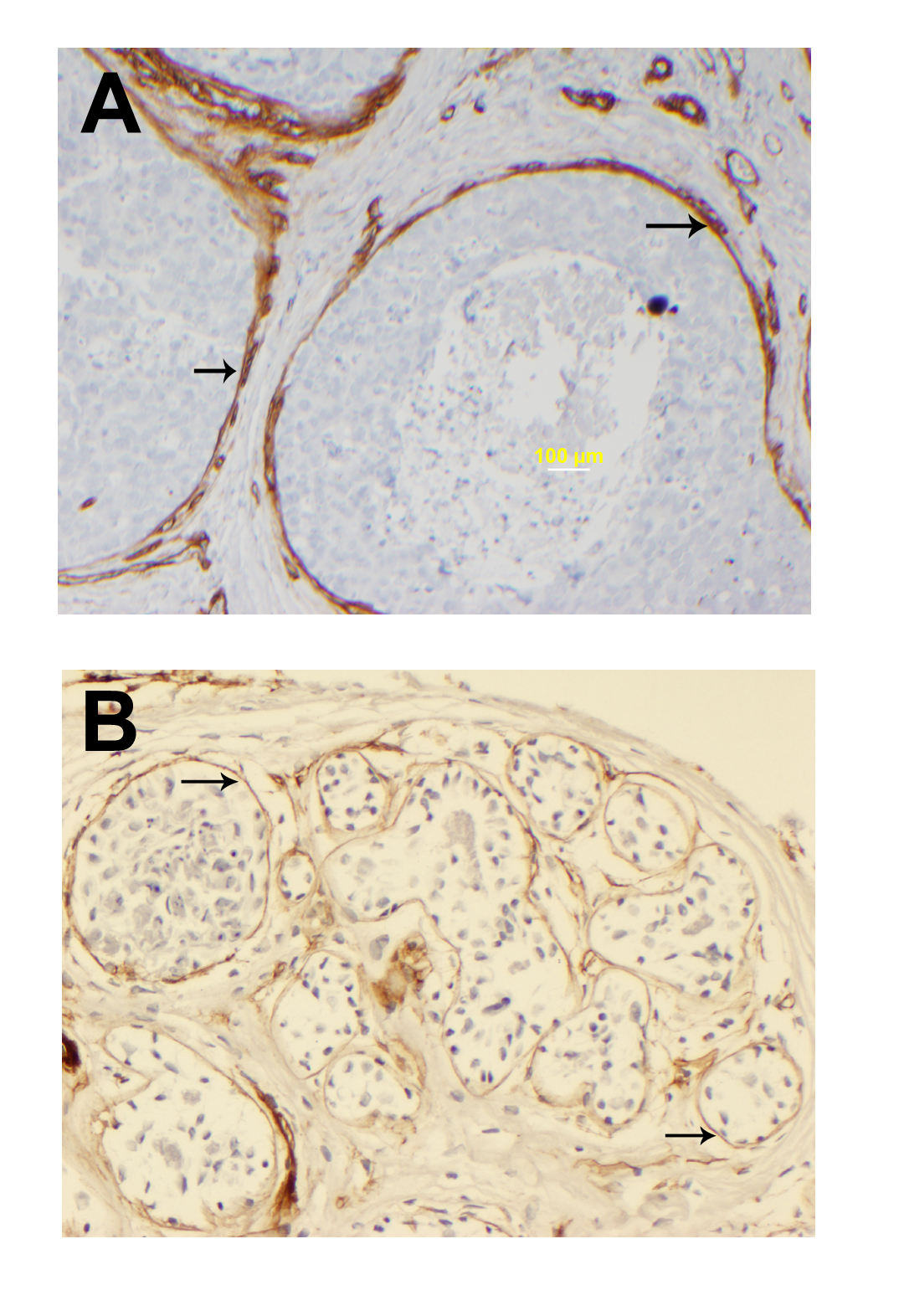

Supplement: Figure S8 — Basement membrane remains intact in DCIS cultured organoids. Collagen type IV immunohistochemistry demonstrates intact basement membrane surrounding breast ducts (brown staining). (A) Case 08-352 FFPE primary breast tissue at time of procurement. Scale bar 100 µm, magnification 10×. (B) The basement membrane remains intact surrounding the duct that is within the tissue fragment and surviving after the breast organoid was growing in culture for 12 weeks (case 08-148, magnification 20×). (2.39 MB TIF) [file pone.0010240.s008.tif]
