# Supplementary material for: Malignant Precursor Cells Pre-Exist in Human Breast DCIS and Require Autophagy for Survival
Source: PLoS One. 2010 Apr 20;5(4):e10240. doi: 10.1371/journal.pone.0010240 (PMC2857649; doi:10.1371/journal.pone.0010240)
Supplement: Table S3 — Primary antibodies and antigen retrieval conditions for immunohistochemistry. (0.03 MB DOC) [file pone.0010240.s011.doc]

| **Antibody** | **Vendor** | **Species** | **Dilution** | **Proteinase K** | **HIER** |
| --- | --- | --- | --- | --- | --- |
| Atg 5 | Abcam | Rabbit | 1:300 | None | pH 6 |
| Atg 7 | Sigma | Rabbit | 1:25 | 5 min | None |
| Beclin-1 | Sigma | Rabbit | 1:750 | 5 min | None |
| CD44 | Cell Signaling Technology | Mouse | 1:50 | 5 min | None |
| Collagen IV | Dako | Mouse | 1:25 | 5 min | pH 6 |
| LC3B | Cell Signaling Technology | Rabbit | 1:25 | None | pH 9 |
| SUPT3H | Abnova | Mouse | 1:50 | 5 min | None |

Table S3. Primary antibodies and antigen retrieval conditions for immunohistochemistry.

HIER=heat induced epitope retrieval; 20 minutes at 95^o^C.
